# Structural dissection of a complex *Bacteroides ovatus* gene locus conferring xyloglucan metabolism in the human gut

**DOI:** 10.1098/rsob.160142

**Published:** 2016-07-27

**Authors:** Glyn R. Hemsworth, Andrew J. Thompson, Judith Stepper, Łukasz F. Sobala, Travis Coyle, Johan Larsbrink, Oliver Spadiut, Ethan D. Goddard-Borger, Keith A. Stubbs, Harry Brumer, Gideon J. Davies

**Affiliations:** 1 Department of Chemistry, York Structural Biology Laboratory, The University of York, Heslington, York YO10 5DD, UK; 2 School of Chemistry and Biochemistry, The University of Western Australia, Crawley, Western Australia 6009, Australia; 3 Division of Glycoscience, School of Biotechnology, Royal Institute of Technology (KTH), AlbaNova University Centre, 106 91 Stockholm, Sweden; 4 Michael Smith Laboratories and Department of Chemistry, University of British Columbia, 2185 East Mall, Vancouver, British Columbia, CanadaV6T 1Z4; 5 Wallenberg Wood Science Center, Royal Institute of Technology (KTH), Teknikringen 56–58, 100 44 Stockholm, Sweden; 6 The Walter and Eliza Hall Institute of Medical Research, 1G Royal Parade, Parkville Victoria 3052, Australia

**Keywords:** xyloglucan, polysaccharide utilization loci, glycoside hydrolases

## Abstract

The human gastrointestinal tract harbours myriad bacterial species, collectively termed the microbiota, that strongly influence human health. Symbiotic members of our microbiota play a pivotal role in the digestion of complex carbohydrates that are otherwise recalcitrant to assimilation. Indeed, the intrinsic human polysaccharide-degrading enzyme repertoire is limited to various starch-based substrates; more complex polysaccharides demand microbial degradation. Select Bacteroidetes are responsible for the degradation of the ubiquitous vegetable xyloglucans (XyGs), through the concerted action of cohorts of enzymes and glycan-binding proteins encoded by specific xyloglucan utilization loci (XyGULs). Extending recent (meta)genomic, transcriptomic and biochemical analyses, significant questions remain regarding the structural biology of the molecular machinery required for XyG saccharification. Here, we reveal the three-dimensional structures of an α-xylosidase, a β-glucosidase, and two α-l-arabinofuranosidases from the *Bacteroides ovatus* XyGUL. Aided by bespoke ligand synthesis, our analyses highlight key adaptations in these enzymes that confer individual specificity for xyloglucan side chains and dictate concerted, stepwise disassembly of xyloglucan oligosaccharides. In harness with our recent structural characterization of the vanguard endo-xyloglucanse and cell-surface glycan-binding proteins, the present analysis provides a near-complete structural view of xyloglucan recognition and catalysis by XyGUL proteins.

## 1. Background

The metabolism of complex carbohydrates in the distal gastrointestinal (GI) tract is central to human nutrition and health [1,2]. It is widely understood that a well-balanced human diet consists of a significant proportion of fruits and vegetables, the cell walls of which are primarily (approx. 90% of the dry weight) comprised of a structurally diverse array of intrinsically non-digestible polysaccharides popularly referred to as ‘dietary fibre’ [1–5]. The human genome is, however, remarkably bereft of genes encoding the enzymes necessary to digest the manifold plant polysaccharides we ingest, with the exception of the α-glucans, amylose and amylopectin, that constitute starch [6]. Even in this case, certain structurally compact, recalcitrant forms, known as ‘resistant starches’ (RS), may reach the colon intact [3]. Both RS and the diverse non-starch polysaccharides (NSP) of plant cell walls are instead metabolized, to various extents, by our symbiotic gut microbiota. Microbial fermentation of monosaccharides in the gut produces short chain fatty acids (SCFAs), which provide a notable proportion (up to 10%) of our daily caloric intake. In addition, localized butyrate production is particularly required to maintain a healthy colonic epithelium [7–9]. There is, therefore, intense current research focus on (and considerable popular interest in) potential causal links between imbalance of the microbiota (dysbiosis) and a wide array of human diseases, including irritable bowel diseases, persistent *Clostridium difficile* infection, metabolic syndrome, diabetes, atopy and neurological disorders [10–14].

Thus, human health is crucially dependent on the population dynamics of the gut ecosystem, which is, in turn, rooted in the capacity of the microbiota to utilize the complex carbohydrates that we are otherwise incapable of accessing [15,16]. Strikingly, many *individual* microbiotal species, especially from the phylum Bacteroidetes, possess the genetic capacity to produce *hundreds* of predicted carbohydrate-active enzymes (CAZymes) [6,17]. This tremendous diversity is directly reflective of the natural structural complexity of plant, fungal and animal oligosaccharides and polysaccharides in the human diet [5,16]. Numerous (meta)genomic, transcriptomic and proteomic studies are continuing to provide a wealth of information on the genetic potential and dynamic response of the human gut microbiome with regard to complex carbohydrate catabolism [9,17–22]. However, our functional understanding of the molecular mechanisms fuelling this ecosystem is currently only in its infancy, due to a comparative paucity of enzymology and structural biology [23,24]. Indeed, among glycoside hydrolases (GH) from all organisms, biochemically and structurally characterized examples total only approximately 5% and 0.5%, respectively, of known open-reading frames (ORFs) [25]; these values are much lower for gut bacterial species.

**Figure 1.**
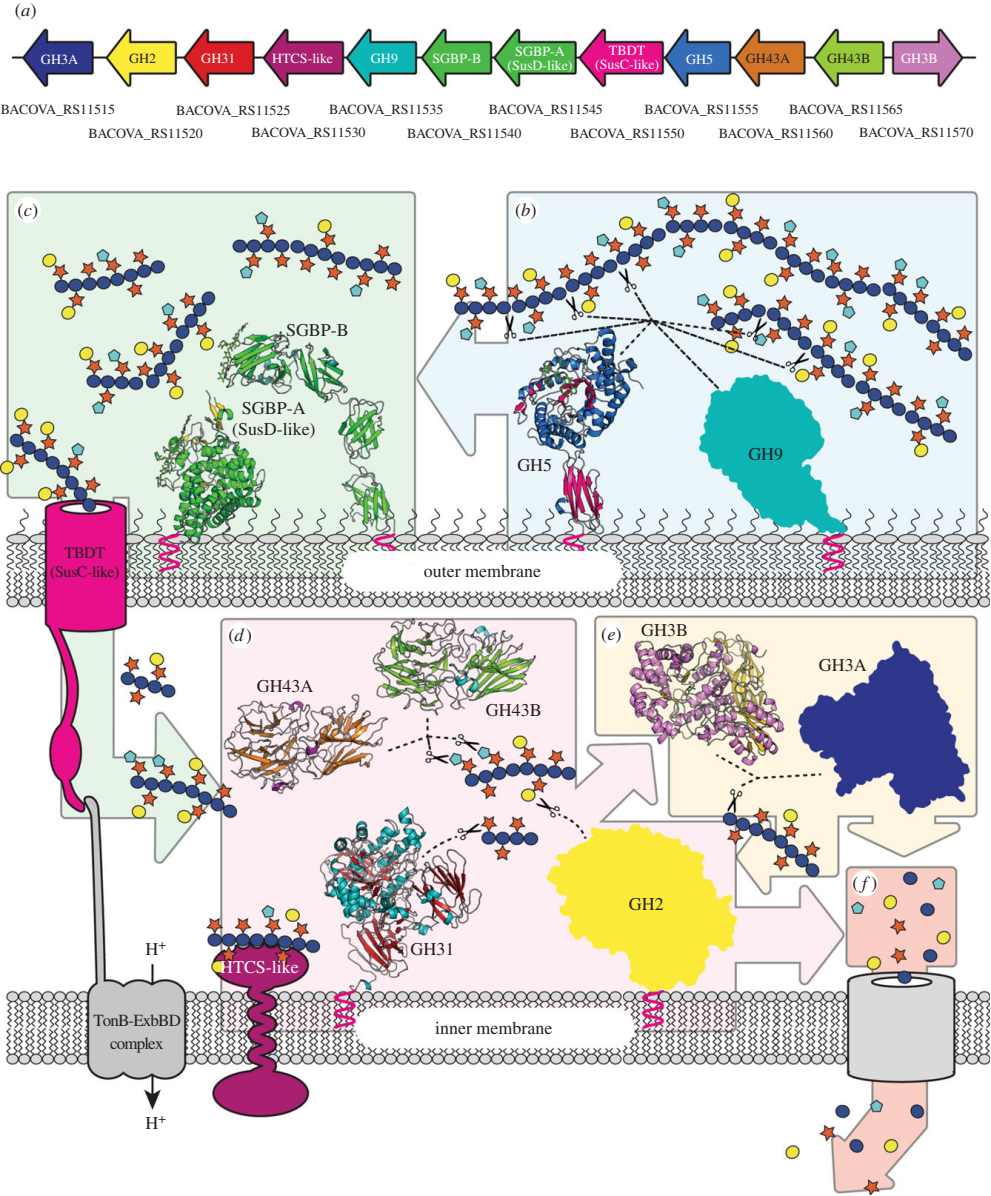
Summary of the xyloglucan saccharification system encoded by the *Bo*XyGUL. (*a*) Gene organization of the *Bo*XyGUL, coloured with reference to the proteins shown in subsequent panels. GenBank locus tag numbers are indicated below each gene. (*b*) *endo*-Xyloglucanases GH5 (structure from [32]) and GH9 localized to the surface of the cell cleave long xyloglucan polysaccharides into smaller fragments, with glycan capture facilitated by cell-surface glycan-binding proteins SGBP-A and SGBP-B (structures from [33]). (*c*) The resulting shorter oligosaccharides are imported into the periplasm via the TonB-dependent transporter (TBDT) for further processing. (*d*) *exo*-Glycosidases GH31, GH43A, GH43B and GH2 remove pendant xylosyl, arabinofuranosyl and galactosyl side chains. (*e*) GH3A and GH3B β-glucosidases act from the non-reducing end liberating individual glucose residues. The oligosaccharides can be further hydrolysed, by these individual enzymes, into monosaccharides. (*f*) The liberated sugars are imported into the cell and metabolized. See [34] for a detailed summary of XyG side-chain structures; monosaccharides are represented using standard Consortium of Functional Glycomics symbols [35].

The two dominant phyla in the colon of healthy adult humans are the Gram-positive Firmicutes and the Gram-negative Bacteroidetes [26], individual species of which have been implicated as key contributors to the breakdown of NSP in the diet [17,19,27,28]. Bacteroidetes are particularly notable for organizing cohorts of CAZymes and binding, transport and sensor/regulator proteins into contiguous polysaccharide utilization loci (PULs) [23,29,30]. Bacteroidetes PUL complexity generally scales with the monosaccharide and linkage complexity of the cognate substrate, especially with regard to the number of GHs and polysaccharide lyases (PLs) [17,19,23]. As such, PULs often encode complete molecular systems for the specific utilization of individual polysaccharides. Likewise, intimate coordination of substrate adherence and initial backbone cleavage at the cell surface, followed by complete oligosaccharide hydrolysis in the confines of the periplasmic space, represents a particularly elegant evolutionary strategy to limit loss of monosaccharides to the competitive gut environment [31] (figure 1).

Transcending ‘omics’ surveys of the gut microbiota, an emerging methodology for the in-depth functional characterization of PULs combines bacterial genetics, biochemistry and enzymology, and structural biology. A growing number of such system-based approaches have been used to elucidate the complex molecular details of fructan [36], seaweed porphyran [37], yeast mannan [38] and cereal xylan [39] utilization by symbiotic human gut *Bacteroides* species. In this context, we recently reported the characterization of a novel xyloglucan utilization locus (XyGUL) that confers *Bacteroides ovatus*, and species harbouring syntenic XyGULs, with the ability to utilize this abundant vegetable polysaccharide across sampled human populations [32]. In this work, the complete biochemical and crystallographic characterization of the vanguard *endo*-xyloglucanase responsible for initiating substrate backbone cleavage was presented, in addition to biochemical data revealing the substrate specificity of the six downstream *exo*-glycosidases. Together, these data allowed us to outline a general pathway for dietary xyloglucan saccharification to monosaccharides for primary metabolism. Until now, however, molecular-level insight into xyloglucan oligosaccharide (XyGO) recognition and hydrolysis by these key downstream enzymes has been lacking. Here, we present the three-dimensional structures of *Bo*GH31, *Bo*GH43A, *Bo*GH43B and *Bo*GH3B, expanding our knowledge of the structural determinants required for xyloglucan degradation (figure 1). Our analyses highlight key adaptations in these enzymes that confer their specificity for xyloglucan oligosaccharides, while also providing a rationale for the maintenance of two divergent genes coding for GH3 enzymes, and similarly two divergent genes for GH43 family members, within the same PUL.

## 2. Material and methods

### 2.1. Cloning, over-expression and structure determination of *Bo*GH31

For structural characterization, the gene encoding *Bo*GH31 was recloned from pET21a(GH31) [32] into a modified pET28a vector (pET-YSBL3C) containing an N-terminal His_6_-tag for immobilized metal affinity purification (IMAC) and 3C-cleavage site to allow subsequent removal of the tag [40]. The GH31 ORF was amplified from the pET21a(GH31) template and cloned into linearized pET-YSBL3C using the InFusion-HD cloning kit (ClonTech), according to the manufacturer's instructions, to give pET-YSBL3C(GH31).

Chemically competent *Escherichia coli* TUNER(DE3) cells were transformed with the pET-YSBL3C(GH31) vector and grown in LB medium containing 50 µg ml^−1^ kanamycin at 37°C. Once the cells reached an OD_600 nm_ of 0.8–1.0, the temperature was lowered to 16°C and expression was induced by the addition of isopropyl β-d-galactopyranoside (IPTG) to a final concentration of 200 µM and the expression was allowed to proceed overnight. Cells were harvested by centrifugation at 10 800*g* for 20 min at 4°C. Spent medium was discarded and the cells were resuspended in 5× volumes of Buffer A (50 mM HEPES pH 7, 0.3 M NaCl, 10 mM imidazole). Cells were lysed with four 20 s pulses of sonication at maximum amplitude in an MSE Soniprep 150 sonicator on ice. Cell debris was removed by centrifugation at 3900*g* in a cooled bench top centrifuge and the cleared lysate was applied directly to a 5 ml HisTrap FF Crude column (GE Healthcare). After washing with 5–6 volumes of Buffer A, protein was eluted with a linear gradient from 0 to 100% Buffer B (50 mM HEPES pH 7, 0.3 M NaCl, 500 mM imidazole) over 20 column volumes, collecting 6 ml fractions. Peak fractions containing *Bo*GH31 were combined and concentrated to less than 2 ml using a 50 kDa cut-off Sartorius concentrator before being applied to a HiTrap 16/60 superdex 200 column (GE Healthcare), which had been equilibrated with 25 mM HEPES pH 7, 100 mM NaCl and 1 mM DTT. After a void volume of 40 ml, 2 ml fractions were collected and those containing *Bo*GH31 were combined and concentrated using a 50 kDa cut-off Sartorius concentrator. Protein concentration was determined to be 35 mg ml^−1^ as judged by A_280 nm_ using an extinction coefficient of 238735 M^−1^ cm^−1^ and a molecular weight of 109 815.6 Da.

Crystals of *Bo*GH31 were obtained by hanging drop vapour diffusion (19°C) using 0.2 M potassium thiocyanate, 14–20% (w/v) PEG-3350 as mother liquor and were used for subsequent structure determination. Crystals were cryo-cooled for data collection at 100 K by plunging in liquid nitrogen after a 30 s soak in mother liquor supplemented with 20% ethylene glycol. Crystals of *Bo*GH31 in complex with a covalent inhibitor, 5FIdoF [41,42], were obtained by soaking native crystals in 10 mM (final) 5FIdoF supplemented with mother liquor for 30 s, immediately prior to cryocooling. Diffraction data for native *Bo*GH31 were collected at Diamond Light Source, beamline i04-1 at a wavelength of 0.920 Å, while data for the covalent 5FIdoF complex were collected at beamline i04 (also Diamond Light Source, *λ* = 0.9795 Å). All data were indexed and integrated using XDS [43] with all subsequent processing steps performed using the CCP4 software suite [44]. The structure was solved by molecular replacement in MOLREP [44] using the protein chain in PDB entry 2xvg as the search model. An initial model was generated using ARP-WARP [45] before subsequent model building and refinement were performed in COOT [46] and REFMAC [47], respectively.

### 2.2. Cloning, over-expression and structure determination of *Bo*GH43A

For structural characterization, the gene encoding *Bo*GH43A was recloned from pET21a [32] into pET28a containing an N-terminal His_6_-tag for IMAC. The *Bo*GH43A ORF was amplified from the pET21a(*Bo*GH43A) template and cloned into linearized pET28a using the InFusion-HD cloning kit (ClonTech) according to the manufacturer's instructions. Protein expression and purification were performed exactly as described above for *Bo*GH31. The final *Bo*GH43A sample was concentrated on a 30 kDa cut-off Sartorius concentrator to 103 mg ml^−1^ as judged by A_280 nm_ using an extinction coefficient of 105 450 M^−1^ cm^−1^ and a molecular weight of 57 965.1 Da.

Crystals of *Bo*GH43A were obtained by hanging drop vapour diffusion (19°C) using 0.1 M Tris pH 7.2–7.8, 0.18 M magnesium chloride and 12% (w/v) PEG-6000 as mother liquor and were used for subsequent structure determination. Crystals were cryo-cooled for data collection at 100 K by plunging in liquid nitrogen after a 30 s soak in mother liquor supplemented with 20% ethylene glycol. Crystals of *Bo*GH43A in complex with AraDNJ and AraLOG were obtained by soaking native crystals in 10 mM (final) solutions of respective compounds supplemented with mother liquor for 60 min, prior to cryocooling. Diffraction data for native *Bo*GH43A were collected at Diamond Light Source, beamline i04-1 at a wavelength of 0.920 Å, while datasets for AraDNJ and AraLOG complexes were both collected at beamline i03 (*λ* = 0.9795 Å). All data were indexed and integrated using XDS [43] with all subsequent processing steps performed using the CCP4 software suite [44]. The structure was solved by molecular replacement in PHASER [48] using the protein chain from previously solved *Bo*GH43B as the search model. An initial model was generated using BUCCANEER [49,50] before subsequent model building and refinement were performed in COOT [46] and REFMAC [47], respectively.

### 2.3. Over-expression and structure determination of *Bo*GH43B

Chemically competent *E. coli* BL21 (DE3) cells were transformed with pET21a(*Bo*GH43B) [32] and grown in LB medium containing 100 µg ml^−1^ ampicillin at 37°C. Once the cells reached an OD_600_ of 0.4–0.6, the temperature was lowered to 16°C and expression was induced by the addition of IPTG to a final concentration of 100 µM and the expression was allowed to proceed overnight. Cells were harvested by centrifugation at 10 800*g* for 20 min at 4°C. Spent medium was discarded and the cells were resuspended in 5× volumes of Buffer A (50 mM HEPES pH 7, 0.5 M NaCl, 30 mM imidazole). Cells were lysed with four 20 s pulses of sonication at maximum amplitude in an MSE Soniprep 150 sonicator on ice. Cell debris was removed by centrifugation at 39 000*g* and the supernatant was applied directly to a 5 ml HisTrap FF Nickel NTA column (GE HEalthcare). After washing with five volumes of Buffer A, protein was eluted with a linear gradient from 0 to 100% Buffer B (50 mM HEPES pH 7, 0.5 M NaCl, 300 mM imidazole) over 20 column volumes, collecting 1.6 ml fractions. Peak fractions containing *Bo*GH43B were combined and concentrated to less than 1 ml using a 30 kDa cut-off Sartorius concentrator before being applied to a HiTrap 16/60 superdex 200 column (GE Healthcare) which had been equilibrated with 10 mM HEPES pH 7, 250 mM NaCl. After a void volume of 40 ml, 1.6 ml fractions were collected and those containing *Bo*GH43B were combined, concentrated and buffer exchanged with 10 mM HEPES pH 7 on a 30 kDa cut-off Sartorius concentrator. Protein concentration was determined to be 10 mg ml^−1^ as judged by *A*_280 nm_ using an extinction coefficient of 102 790 M^−1^ cm^−1^ and a molecular weight of 57 243.3 Da.

Crystals of *Bo*GH43B were obtained by hanging drop vapour diffusion using 0.2 M sodium acetate pH 5, 20–30% PEG-3350 as mother liquor and they were used for subsequent structure determination. Crystals were cryo-cooled for data collection at 100 K by plunging in liquid nitrogen after a 30 s soak in mother liquor supplemented with 20% ethylene glycol. Diffraction data were collected at Diamond Light Source, beamline i02 at a wavelength of 0.980 Å. The data were indexed and integrated using XDS [43] with all subsequent processing steps performed using the CCP4 software suite [44]. The structure was solved by molecular replacement in PHASER [48] using the protein chain in PDB entry 1yrz as the search model. The initial phases were improved using PARROT [51] and an initial model generated using BUCCANEER [49,50] before subsequent model building and refinement were performed in COOT [46] and REFMAC [47], respectively.

### 2.4. Over-expression and structure determination of GH3B

GH3B expression and purification from the pET21a(GH3B) construct created by Larsbrink *et al*. [32] was performed as described above for *Bo*GH43B. The final sample was prepared at 10 mg ml^−1^ as judged by the *A*_280 nm_ using an extinction coefficient of 142 670 M^−1^ cm^−1^ and a molecular weight of 86 512.6 Da.

Crystals were obtained by hanging drop vapour diffusion using 0.2 M sodium acetate and 15–25% PEG-3350 as the mother liquor. Crystals were cryo-cooled by plunging in liquid nitrogen using mother liquor supplemented with 20% ethylene glycol as the cryo-protectant prior to data collection at Diamond Light Source, beamline i04-1 at a wavelength of 0.920 Å. Indexing and integration of diffraction data was performed with XDS [43] with all subsequent data processing performed using the CCP4 software suite [44]. Data were phased by molecular replacement in PHASER [48] using the barley β-glucosidase structure 1ex1 [52] as the search model. Phase improvement was performed using PARROT [51] before generation of an initial model using BUCCANEER [49,50]. Subsequent model building and refinement were performed in COOT [46] and REFMAC [47], respectively. TLS refinement using two TLS groups per protein chain was invoked towards the end of structure refinement.

### 2.5. Synthesis of arabinofuranosidase inhibitors

#### 2.5.1. General

^1^H and ^13^C nuclear magnetic resonance spectra were obtained on Bruker ARX500 (500 MHz for ^1^H and 125 MHz for ^13^C) or Bruker AV600 (600 MHz for ^1^H and 150 MHz for ^13^C) spectrometers (see the electronic supplementary material). Mass spectra were recorded with a Waters GCT Premier spectrometer using electrospray ionization (ES).

#### 2.5.2. (*E*) and (*Z*)-2,3,5-Tri-*O*-acetyl-l-arabinofuranose oxime (**2**)

Hydroxylamine hydrochloride (240 mg, 3.45 mmol) was added to a solution of the hemiacetal **1** [53] (610 mg, 2.21 mmol) and pyridine (0.45 ml, 5.5 mmol) in MeOH (20 ml) and the mixture was stirred at reflux (2 h). Concentration of the solution by co-evaporation with toluene (3 × 15 ml) followed by flash chromatography of the residue (6 : 4 EtOAc/hexanes) produced the presumed oxime **2** as a white solid (575 mg, 94%). *R_f_* 0.40 (7 : 3 EtOAc/hexanes). This solid was used without further purification.

#### 2.5.3. (*Z*)-2,3,5-Tri-*O*-acetyl-l-arabinonhydroximo-1,4-lactone (**3**)

1,8-Diazabicyclo[5.4.0]undec-7-ene (0.35 ml, 2.3 mmol) was added to a solution of the oxime **2** (575 mg, 2.08 mmol) and NCS (305 mg, 2.28 mmol) in CH_2_Cl_2_ (21 ml) at −40°C, in such a way that the temperature did not rise above −35°C, and the resulting mixture was stirred at −40°C for 1 h before being allowed to warm to room temperature over 2 h. The resulting solution was quenched with water and diluted with CH_2_Cl_2_ (20 ml). The organic layer was separated and washed with water (3 × 15 ml), brine, dried (MgSO_4_), filtered and concentrated. Flash chromatography of the residue (3 : 2 EtOAc/hexanes) yielded the triacetate **3** as a colourless oil (410 mg, 71%). *R_f_* 0.38 (7 : 3 EtOAc/hexanes). ^1^H NMR (500 MHz, CDCl_3_): *δ* 6.96 (*br* s, 1H), 5.74 (d, 1H, *J* = 2.8 Hz), 5.22–5.20 (m, 1H), 4.68–4.63 (m, 1H), 4.42 (dd, 1H, *J* = 4.5, 12.0 Hz), 4.31 (dd, 1H, *J* = 6.0, 12.0 Hz), 2.15 (s, 3H), 2.13–2.11 (m, 6H); ^13^C NMR (125 MHz, CDCl_3_): *δ* 170.66, 169.89, 169.28, 154.26, 83.37, 74.90, 72.46, 62.39, 20.69, 20.65. HRMS (ES): *m*/*z* = 312.0683; [M + Na]^+^ requires 312.0695.

#### 2.5.4. (*Z*)-l-Arabinonhydroximo-1,4-lactone (AraLOG)

Saturated ammonia in MeOH (5 ml) was added to a solution of the triacetate **3** (100 mg, 0.346 mmol) in MeOH (5 ml) at 0°C and the solution was allowed to stand (0°C, 2 h). Concentration of the solution followed by flash chromatography of the residue (3 : 7 MeOH/EtOAc) yielded the title compound (39 mg, 68%). *R_f_* 0.37 (3 : 7 MeOH/EtOAc). ^1^H NMR (600 MHz, D_2_O): *δ* 4.70 (d, 1H, *J* = 7.2 Hz), 4.39–4.33 (m, 1H), 4.20 (dd, 1H, *J* = 7.2 Hz), 4.00–3.95 (m, 1H), 3.81 (dd, 1H, *J* = 4.8, 13.2 Hz); ^13^C NMR (150 MHz, D_2_O): *δ* 159.00, 84.79, 73.95, 73.71, 59.99. HRMS (ES): *m*/*z* = 164.0551; [M + H]^+^ requires 164.0559.

#### 2.5.5. (*Z*)-*O*-(2,3,5-Tri-*O*-acetyl-L-arabinosylidene)amino *N*-phenylcarbamate (**4**)

Phenyl isocyanate (50 µl, 0.46 mmol) was added to a solution of the triacetate **3** (105 mg, 0.363 mmol) and Et_3_N (0.16 ml, 1.2 mmol) in THF (5 ml) at 0°C and the solution was stirred (0°C, 2 h). Concentration followed by flash chromatography of the residue (1 : 1 EtOAc/hexanes) produced the carbamate **4** as a colourless foam (90 mg, 57%). *R_f_* 0.31 (1 : 1 EtOAc/hexanes). ^1^H NMR (500 MHz, CDCl_3_): *δ* 7.76 (*br* s, 1H), 7.49–7.44 (m, 2H), 7.36–7.30 (m, 2H), 7.14–7.09 (m, 1H), 5.86 (d, 1H, *J* = 3.0), 5.24 (dd, 1H, *J* = 2.5, 3.0 Hz), 4.77–4.74 (m, 1H), 4.46 (dd, 1H, *J* = 4.5, 12.5 Hz), 4.34 (dd, 1H, *J* = 6.0, 12.5 Hz), 2.20 (s, 3H), 2.15 (s, 3H), 2.14 (s, 3H); ^13^C NMR (125 MHz, CDCl_3_): *δ* 170.38, 169.70, 168.88, 157.69, 151.21, 137.00, 129.07, 124.14, 119.36, 85.25, 77.16, 74.70, 72.85, 62.02, 20.60, 20.52. HRMS (ES): *m*/*z* = 409.1248; [M + H]^+^ requires 409.1247.

#### 2.5.6. (*Z*)-*O*-(L-Arabinosylidene)amino *N*-phenylcarbamate (AraPUG)

Saturated ammonia in MeOH (5 ml) was added to a solution of the carbamate **4** (80 mg, 0.20 mmol) in MeOH (5 ml) at 0°C and the solution was allowed to stand (0°C, 2 h). The resulting solution was concentrated to yield a white solid. Trituration of the solid (1 : 4 : 95 H_2_O/MeOH/EtOAc) yielded the title compound as a white powder (43 mg, 78%). *R_f_* 0.26 (1 : 9 MeOH/EtOAc). ^1^H NMR (600 MHz, (CD_3_)_2_SO): *δ* 9.78 (*br* s, 1H), 7.52–7.47 (m, 2H), 7.32–7.26 (m, 2H), 7.03–6.99 (m, 1H), 6.21 (*br* s, 1H), 5.85 (*br* s, 1H), 5.14 (*br* s, 1H), 4.46 (d, 1H, *J* = 4.8 Hz), 4.26–4.22 (m, 1H), 4.01 (m, 1H), 3.71 (m, 1H), 3.58 (m, 1H); ^13^C NMR (150 MHz, (CD_3_)_2_SO): *δ* 163.17, 151.81, 138.71, 128.75, 122.71, 118.58, 88.38, 74.45, 73.77, 59.91. HRMS (ES): *m*/*z* = 283.0928; [M + H]^+^ requires 283.0930.

### 2.6. Binding constant determination for AraF inhibitors

Binding of two arabinofuranosidase inhibitors, AraDNJ and AraLOG, to *Bo*GH43A and *Bo*GH43B was investigated by isothermal titration calorimetry (ITC) in a MicroCal Auto-ITC200 system (GE Healthcare/Malvern Instruments). *Bo*GH43A titrations were performed in 25 mM HEPES pH 7.0, 100 mM NaCl and 1 mM DTT, while *Bo*GH43B titrations used 25 mM HEPES pH 7.0, 100 mM NaCl. Ligands were prepared by dilution in the identical buffer as used for protein sample preparation. AraLOG binding could not be detected to either *Bo*GH43A or B with titrations performed in triplicate at 25°C, with 1 mM AraLOG titrated into 100 µM pure protein. An interaction between AraDNJ and both proteins, however, could be detected but appeared to be weak and so low c-value ITCs were performed to obtain binding data [54]. Assays were conducted in triplicate at 25°C, with 2 mM AraDNJ titrated into approximately 100 µM protein (more precise protein concentrations were measured for each sample immediately before performing the titrations and these values were used for data fitting in Origin). To obtain saturation, titrations were split into two runs, the first consisting of a single 1 µl injection at the start of the run (discarded during the analysis) followed by 19× 2 µl injections of ligand. At the end of this run 39 µl was removed from the cell, the syringe was refilled with ligand and the titration was continued with 20 additional 2 µl injections. CONCAT32 (MicroCal) was then used to concatenate the data together into a single titration. To account for heats of dilution, an additional titration was performed in exactly the same way, titrating ligand into buffer. These reference data were then subtracted from all experimental data which were subsequently used to calculate dissociation constants (*K*_d_) using the Origin 7 software package by fixing the *N*-value at 1.0 during the fitting (MicroCal, see figure 3*d*).

## 3. Results and discussion

### 3.1. Structure of the α-xylosidase *Bo*GH31

As with many of the glycoside hydrolase families represented within the *Bo* xyloglucan PUL (XyGUL), GH31 forms a large (currently over 3800 sequences) and functionally diverse collection of enzymes, with many α-glucosidases, α-xylosidases and α-galactosidases featuring prominently [25]. Within XyGULs, GH31 α-xylosidases play an essential role removing xylose from the non-reducing end of processed xyloglucan oligosaccharides (illustrated in figure 1*d*). Such activity permits enzymatic access to the β-1,4-linked glucose moieties of the XyGO backbone. Indeed, deletion of the gene encoding GH31 from the XyGUL completely abrogates the ability of *B. ovatus* to grow on XyG and XyGOs [32]. Consistent with this role, the GH31 α-xylosidase present within the *Bo* XyGUL (*Bo*GH31) has been shown to be highly active against native XyGO substrates (XXXG and XLLG, nomenclature according to [34]), rather than disaccharide-configured activity probes, such as Xyl-α-PNP [32], despite the presence of optimized chemical leaving groups requiring little protonic assistance from the enzyme. These observations suggest substrate binding by XyGO-active GH31 enzymes to be a both complex and highly specific process, requiring recognition and occupancy of multiple sub-sites distal to the catalytic centre.

**Figure 2.**
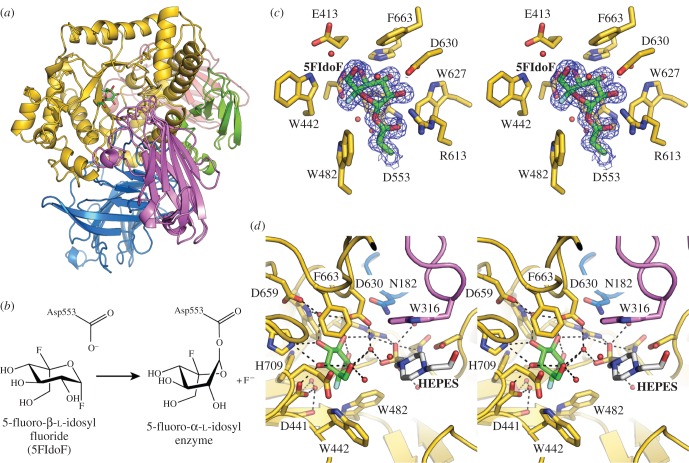
*Bo*GH31 α-xylosidase overall and active site structure. (*a*) Overall structure of *Bo*GH31 coloured by domain: N-terminal β sandwich in blue, the PA14 domain in purple, TIM barrel domain in gold, C-terminal proximal β-sandwich in green and C-terminal distal β-sandwich in red. The location of the active site revealed by the covalent glycosyl-enzyme intermediate is shown as sticks coloured by atom type with green carbons. (*b*) Mechanism of formation of the glycosyl-enzyme intermediate for the *Bo*GH31–5FIdoF complex. (*c*) Wall-eyed stereo view of the active site pocket. The active site nucleophile (Asp553) and 5FIdoF are coloured with green carbon atoms, with the surrounding active site side chains shown with gold carbon atoms. (*d*) Wall-eyed stereo view of the wider active site with the additional fortuitous HEPES molecule (white carbon atoms) shown revealing the likely role of the PA14 domain (purple) in extending the active site pocket to allow binding of longer xyloglucan oligosaccharides.

The crystal structure of *Bo*GH31 was determined to a resolution of 1.5 Å by molecular replacement using the coordinates of *Cj*Xyl31A, a functional homologue present in *Cellvibrio japonicus* (PDB ID: 2xvg, see [55]), as the search model (for X-ray data collection and refinement statistics, see the electronic supplementary material, table S1). A structural comparison of the refined *Bo*GH31 atomic model using PDBeFold [56] revealed close similarity to several other GH31 enzymes, including YicI from *E, coli* (currently the only other structurally characterized α-xylosidase [57]). However, by far the closest structural match to *Bo*GH31 was *Cj*Xyl31A (*Z* score = 33.1, with RMSD = 1.15 Å across 888 matched Cα positions). As observed for *Cj*Xyl31A, *Bo*GH31 presents with an extensive, modular structure featuring several accessory domains appended to a well-conserved TIM barrel-like structure (figure 2*a*) (for a full description of terms and domain nomenclature see [55]). The catalytic core of *Bo*GH31 is composed of residues 384 to 758, which form the central (β/α)_8_ (TIM) barrel fold and harbour the active site (discussed below). The domains decorating the central catalytic unit include an N-terminal β-sandwich domain formed by residues 16 to 213 with additional strands contributed by residues 363 to 383 when the peptide chain returns from a PA14 domain (residues 214 to 362). The presence of PA14 has been observed previously for GH31 in *Cj*Xyl31A and is believed to contribute to the recognition and binding of extended XyGO substrates, as was indicated by NMR spectroscopy and molecular docking studies [55,58]. C-terminal to the central catalytic unit, are two additional domains—the C-terminal proximal (residues 759–839) and distal (residues 840–954) β-sandwiches. While these accessory regions can be thought of as distinct subdomains, extensive interactions and packing of secondary structure elements against the central (β/α)_8_ barrel are strongly suggestive of a low-flexibility, monolithic structure.

The location of the *Bo*GH31 active site and identity of the catalytic amino acids were confirmed through analysis of a covalent enzyme-glycoside intermediate formed between crystals of native *Bo*GH31 and a nucleophile-trapping glycosyl fluoride, 5-fluoro-β-l-idosyl fluoride (5FIdoF) (figure 2*a–c*). Within the complex structure, 5FIdoF forms an α-glycosidic linkage to the side-chain carboxylate of Asp553 at the centre of the (β/α)_8_ barrel. 5FIdoF makes H-bonding interactions to Asp553, Arg613, Asp630 (O2 of the sugar ring), His709 and a highly coordinated water molecule positioned between Asp630 and Asp659 (O3) and Asp441 (O4 and the axially positioned F5 atom). Interestingly, the enzyme-bound 5FIdoF shows significant distortion away from the ^1^*C*_4_ ground state expected for L-sugars, appearing in a ^1^*S*_3_ conformation. Such a conformation is also reflected in various other covalent intermediates with GH31 enzymes, including *Cj*Xyl31A in complex with 5-fluoro-α-d-xylosyl fluoride (5FXylF; also ^1^*S*_3_, see 2xvk [55]) and *Cj*Agd31B, a GH31 α-1,4-transglucosylase, in complex with 5-fluoro-α-d-glucosyl fluoride (5FGlcF; ligand appears midway between ^4^*C*_1_ and ^1^*S*_3_, see 4ba0 [59]).

The *Bo*GH31 covalent glycosyl-enzyme intermediate structure lends further support to the role of the PA14 domain in ligand binding [55]. This domain is in close proximity to the enzyme-bound 5FIdoF, with the side chain of Trp316 approximately 6.5 Å from the ligand (figure 2*d*). Furthermore, a fortuitously bound HEPES molecule, present in the protein buffer, can also be observed in the active site pocket below the plane of the 5FIdoF sugar ring and bridging the gap between ligand and PA14 (figure 2*c*). Within xyloglucan from both dicot and solanaceous species, side-chain xylose moieties are linked α-1,6 to the glucan backbone. Thus backbone sugars occupying the +1, and other potential positive sub-sites, would also highly likely be coordinated below the plane of a −1 xyloside, extending across and out of the catalytic (β/α)_8_ barrel. The positioning of HEPES therefore appears prescient, with the piperazine ring of the ligand engaged in a van der Waals' stacking interaction with Trp513 (catalytic domain) from above, and Trp316 of PA14 from below. The positioning of these aromatic side chains, in addition to numerous other amino acids capable of forming hydrogen bonds, is highly suggestive of a carbohydrate-binding motif, and therefore a direct role for PA14 in the coordination of extended XyGO substrates. A homologous role was proposed for the PA14 domain in the structurally similar, XyGO-specific *Cj*Xyl31A from the saprophyte *C. japonicus* [55,58].

### 3.2. Structures of the α-l-arabinofuranosidases *Bo*GH43A and *Bo*GH43B

GH43 is a large and diverse family of CAZymes with members identified with β-xylosidase, α-l-arabinofuranosidase, arabinanase, xylanase, galactan 1,3-β-galactosidase, α-1,2-l-arabinofuranosidase, exo-α-1,5-l-arabinofuranosidase, exo-α-1,5-l-arabinanase and β-1,3-xylosidase activities. There are two GH43 family members represented in the *B. ovatus* xyloglucan PUL: *Bo*GH43A and *Bo*GH43B [32]. Both enzymes have demonstrable activity on l-Araf-α-PNP, though *Bo*GH43A was considerably more active, and both are thought to be responsible for the removal of pendant arabinofuranoside side chains from solanaceous xyloglucan substrates, thereby converting S to X for further processing by the α-xylosidase and other members of the PUL [32].

#### 3.2.1. Synthesis of arabinofuranosidase inhibitors

**Scheme 1.**
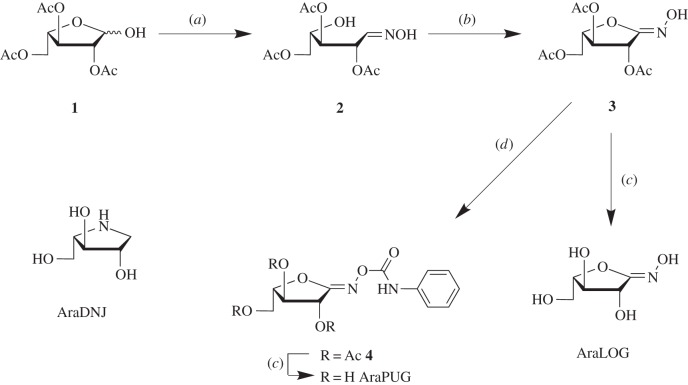
Synthesis of the putative α-l-arabinofuranosidase inhibitors AraLOG and AraPUG as well as the structure of the iminosugar AraDNJ. (*a*) HONH_2_·HCl, C_5_H_5_N, MeOH; (*b*) NCS, DBU, CH_2_Cl_2_; (*c*) NH_3_/MeOH (*d*) PhNCO, Et_3_N, THF.

To aid in the structural characterization of the *Bo*GH43A and *Bo*GH43B active sites, two new potential inhibitors for these enzymes were synthesized. The compounds were prepared incorporating an *sp*^2^-hybridized carbon at carbon-1, which is thought to allow the carbohydrate ring to potentially adopt a conformation that is similar to the geometry of the transition state of glycosidase-catalysed reactions [60]. The synthesis of these inhibitors proceeded from the hemiacetal **1** (scheme 1) [53]. Treatment of the hemiacetal with hydroxylamine hydrochloride yielded the presumed mixture of oximes **2**, which were used without purification and converted to the hydroximolactone **3** in good overall yield. The inhibitor AraLOG was then prepared by treating **3** with saturated ammonia in methanol. Taking the hydroximolactone **3** and treating with phenyl isocyanate furnished the phenyl carbamate **4**. Deprotection of the carbamate **4** under similar conditions used to prepare AraLOG gave AraPUG in good yield.

#### 3.2.2. *Bo*GH43A structure

**Figure 3.**
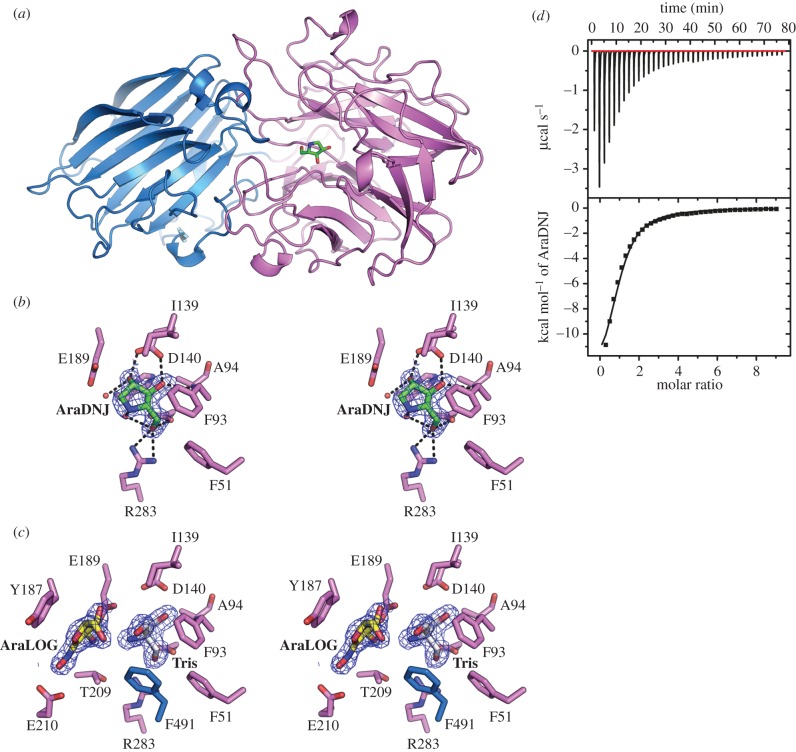
Overall structure and inhibitor binding to *Bo*GH43A α-l-arabinofuranosidase. (*a*) Overall structure of *Bo*GH43A; the N-terminal catalytic domain is coloured purple and C-terminal β-sandwich domain is coloured blue. The location of the active site is indicated by the position of AraDNJ shown in stick representation coloured by atom type with green carbons. (*b*) Wall-eyed stereo view of the active site in the *Bo*GH43A-AraDNJ complex. The final 2Fo-Fc map for the ligand is shown contoured at 1*σ* in blue. The hydrogen bonding interactions made by the inhibitor are shown as black dashed lines. (*c*) Wall-eyed stereo view of the active site for *Bo*GH43A-AraLOG complex. Binding of the AraLOG inhibitor (yellow carbon atoms) was too weak to displace TRIS (white carbon atoms) from the −1 sub-site and instead occupies +1, revealing key stacking interactions with Tyr187 and other conserved residues. (*d*) ITC thermogram showing the binding of AraDNJ to *Bo*GH43A in solution giving a *K*_d_ of 35 ± 4 µM.

The structure of *Bo*GH43A was determined to be 1.6 Å by molecular replacement using the structure of *Bo*GH43B described below as the search model (for X-ray data collection and refinement statistics, see the electronic supplementary material, table S2). Typical of all GH43s, *Bo*GH43A has a two-domain architecture, consisting of an N-terminal 5-bladed β-propeller domain (residues 21 to 321) harbouring the catalytic active site, and a C-terminal β-sandwich domain (residues 322 to 522) which is frequently observed, though can be replaced by carbohydrate binding modules in some family members (see [61] for example) (figure 3*a*). Structural comparisons using PDBeFold [56] reveal close overall matches to other GH43s including XynB from *Bacillus subtilis* subsp. subtilis strain 168 (*Bs*XynB, 1yif; *Z* score = 17.8, with RMSD = 1.44 Å across 478 matched C_α_ positions) and XynB from *Bacillus halodurans* C-125 (*Bh*XynB, 1yrz; *Z* score = 17.7, RMSD = 1.45 Å across 473 matched C_α_s), which all share the same two-domain architecture.

Within the native *Bo*GH43A structure, a TRIS molecule from the crystallization solution was observed bound in a shallow, enclosed pocket proposed to form the *Bo*GH43A −1 sub-site. Soaking of native *Bo*GH43A with two putative inhibitors, AraDNJ [62] and AraLOG, yielded respective enzyme–ligand complexes, confirming this as the active site (figure 3*b*). Disappointingly, no complexes were obtained with AraPUG, despite the use of high concentrations of inhibitor. AraDNJ was able to displace TRIS from the −1 sub-site and appeared bound in a low-energy ^3^*E* conformation typical of iminosugar ‘furanose’ inhibitors. The side-chain carboxylate of Asp140 (O3 and O4 positions), the backbone amino group of Ala94 (O4) and the OD2 atom of Asp34 all directly coordinated the inhibitor (figure 3*b*). GH43 members typically contain three highly conserved acidic residues in their active sites to impart activity [63]. Together with Asp34 as the general base, which activates water to attack the anomeric carbon, Glu189 is ideally poised as the general acid, while Asp140 completes the triplet of residues and is important for modulating the p*K*_a_ and orienting the general acid for catalysis. The positions of these residues are absolutely conserved with other GH43 members.

For the AraLOG complex, repeated soaking at concentrations of up to 25 mM AraLOG for several hours failed to displace TRIS from the −1 sub-site. Rather, AraLOG was instead observed at the +1 site, which would normally be occupied by xylose moieties in the XyGO substrate (figure 3*c*). The AraLOG complex therefore highlights key interactions at this +1 sub-site, with the inhibitor stacking against Tyr187 while also H-bonding directly to the side chains of Glu210 and Glu189. In the light of the inability of AraLOG to displace TRIS from the active site, ITC (in the absence of Tris) was used to probe the affinity of both *Bo*GH43A and *Bo*GH43B (discussed below) for these inhibitors. AraDNJ binds to *Bo*GH43A with *K*_d_ = 35 ± 4 µM (figure 3*d*), while AraLOG binding was too weak to be measured using this technique, consistent with its inability to displace TRIS during crystal soaking.

#### 3.2.3. *Bo*GH43B structure

**Figure 4.**
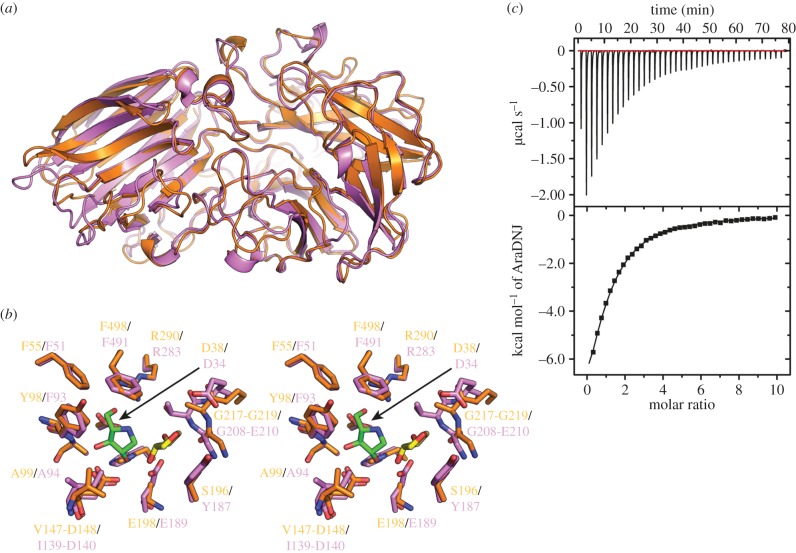
Structural comparison of *Bo*GH43B with *Bo*GH43A. (*a*) Overall superposition of *Bo*GH43B (orange) with *Bo*GH43A (purple) revealing a highly conserved tertiary structure arrangement between the two proteins, even though they share only 41% sequence identity. The largest differences between the two proteins are restricted to altered positioning of loops linking secondary structure elements. (*b*) Comparison of the arrangement of the active site residues between *Bo*GH43B (orange carbons) and *Bo*GH43A (purple carbons). The positions of AraDNJ (green carbons) and AraLOG (yellow carbons) from the *Bo*GH43A structure are shown. The −1 sub-site is mainly conserved between the two proteins, but the +1 sub-site surrounding the AraLOG ligand is significantly different between *Bo*GH43A and B, most notably Tyr187, which stacks against AraLOG in *Bo*GH43A, is replaced by Ser196 in *Bo*GH43B. (*c*) Representative ITC thermogram for the measurement of AraDNJ binding to *Bo*GH43B. Weaker binding was observed to this enzyme compared to *Bo*GH43A giving a *K*_d_ of 111 ± 6 µM, providing a rationale for our inability to obtain a *Bo*GH43B-AraDNJ complex structure.

Despite significant functional overlap with *Bo*GH43A, *Bo*GH43B, the second α-l-arabinofuranosidase present in the *Bo*XyGUL, shares just 41% sequence identity with *Bo*GH43A and appears to be significantly less active on the substrates tested [32]. The structure of *Bo*GH43B was determined to 2.3 Å resolution by molecular replacement using a β-1,4-xylosidase from *B. halodurans* (PDB ID 1yrz) as the search model (electronic supplementary material, table S3). Remarkably, given their apparent differences at the amino acid level, the structure of *Bo*GH43B appears extremely similar to that of *Bo*GH43A, which can be superimposed onto *Bo*GH43B, using GESAMT [44], with an RMSD of 1.24 Å over 482 amino acid residues (figure 4*a*). Comparison of tertiary folds reveals few significant differences between the two paralogues, with the most obvious being the presence of a metal binding site, occupied by calcium, towards the C-terminus of *Bo*GH43B. Such an equivalent site appears entirely absent within *Bo*GH43A. In some GH43 members, addition of divalent cations within the catalytic site has led to increased activity and stability for these enzymes [64–66]. However, the Ca^2+^-binding site in *Bo*GH43B is located in the C-terminal β-sandwich domain, on the opposite side of the molecule from the active site, and similar sites in other family members have not been implicated in catalysis to date [63].

Attempts to obtain structures of *Bo*GH43B in complex with the same inhibitors used for *Bo*GH43A were unsuccessful. ITC was used to determine the affinity of *Bo*GH43B for AraDNJ and AraLOG. *Bo*GH43B bound AraDNJ with a *K*_d_ of 111 ± 6 µM (figure 4*c*), while the affinity for AraLOG was too weak to be measured, as observed for *Bo*GH43A. This weaker binding affinity for AraDNJ also appears consistent with the lower specific activity of *Bo*GH43B for xyloglucan oligosaccharides when compared to its counterpart [32]. Superposition of apo-*Bo*GH43B with AraDNJ-*Bo*GH43A reveals that the three residues implicated in catalysis (Asp38, Asp148 and Glu198 in *Bo*GH43B) are absolutely conserved. The only difference in the *Bo*GH43B −1 sub-site is the replacement of Phe93 (in *Bo*GH43A) with a tyrosine residue in *Bo*GH43B. The +1 sub-site occupied by AraLOG in *Bo*GH43A, however, is considerably different. AraLOG stacks against Tyr187 in *Bo*GH43A, which is replaced by Ser196 in *Bo*GH43B. This variation means the active site pocket in *Bo*GH43B is considerably more open than in its XyGUL paralogue, possibly resulting in weaker substrate binding affinity and hence lower specific activity against authentic XyGO substrates. The reasoning that *B. ovatus* should harbour two GH43 members in its XyGUL remains unclear, but the differences in the active site architecture away from the −1 sub-site may represent the adaptation of these enzymes to specific substrate sources, possibly with alternate Ara*f* structures on XyG branch termini [34].

### 3.3. Structure of β-glucosidase *Bo*GH3B

GH3 represents a large family of over 8000 sequences in the CAZy database. Like GH43, there are two GH3 members (*Bo*GH3A and *Bo*GH3B) present in the *Bo* XyGUL, both of which have been shown to be β-glucosidases with very similar specific activities. Despite apparently duplicated biochemical function, the two enzymes appear to have diverged significantly, sharing only 27% sequence identity at the amino acid level [32]. As for the GH43 enzymes, the functional significance of maintaining two seemingly identical β-glucosidases remains unclear, and so we aimed to structurally characterize both orthologues.

**Figure 5.**
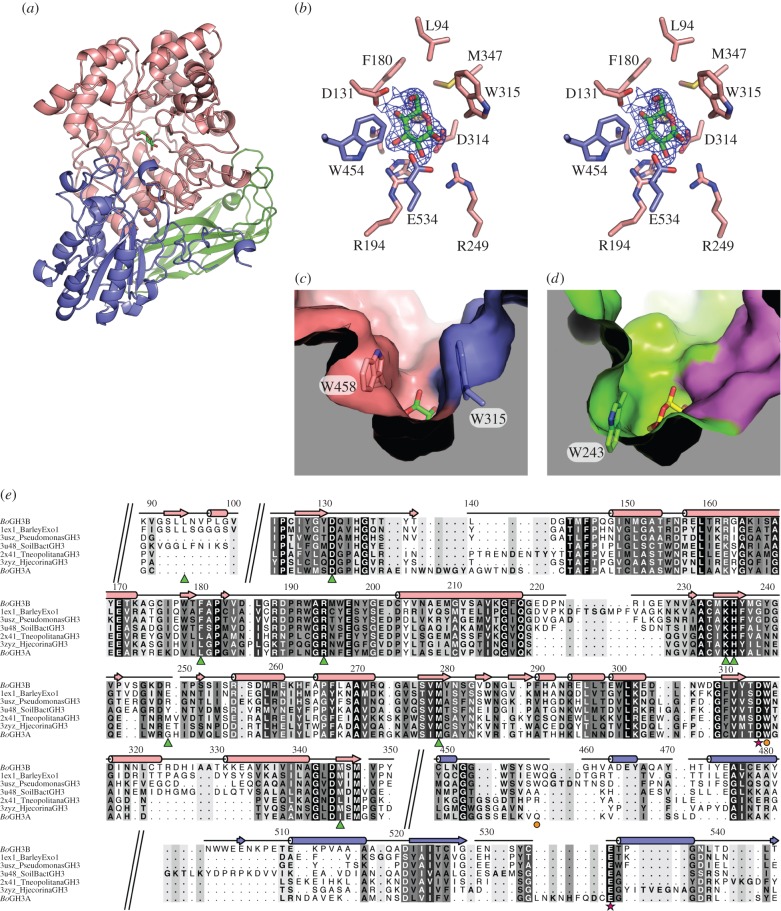
Structural analysis of *Bo*GH3B. (*a*) Overall structure of *Bo*GH3B with the N-terminal TIM-barrel-like domain coloured pink, the α/β sandwich domain coloured purple and the C-terminal FN-III like domain coloured green. The position of the active site is indicated by the presence of glucose, represented as sticks with green carbon atoms. (*b*) Wall-eyed stereo view of the active site in *Bo*GH3B. The final 2Fo-Fc map is shown contoured at 1*σ* as a blue mesh around the glucose molecule that co-purified with the protein. All side chains within 4 Å of glucose are shown as sticks with the carbons coloured according to the domain from which they are provided. (*c*) Surface representation of *Bo*GH3B around the active site pocket revealing a tight entry to the active site resulting from the presence of Trp315 and Trp458, the positions of which are indicated in stick representation. The surface is coloured by domain as for the previous panels and the surface has been clipped for clarity. See the electronic supplementary material, figure S1*a*, for stereo view. (*d*) Surface representation of the active site pocket from *T. neopolitana* GH3 (PDB 2x41), shown from the same perspective as for *Bo*GH3B in (*c*). The surface is coloured with the TIM-barrel domain shown in green and the α/β domain coloured magenta. This enzyme lacks an equivalent residue to Trp458, and the side chain of Trp243 (shown in stick representation) is positioned differently giving a much more open structure to the active site entrance. See the electronic supplementary material, figure S1*b*, for stereo view. (*e*) Structure-based sequence alignment of *Bo*GH3B with other GH3 family members identified through PDBeFold [56]—sections of the alignment have been removed for brevity and breaks are indicated by diagonal double lines across the alignment (the full sequence alignment can be found in the electronic supplementary material, figure S2). The sequence for *Bo*GH3A was added to the structural alignment using MAFFT [67]. The secondary structure elements (coloured by domain as previously) and residue numbers from *Bo*GH3B are indicated along the top of the alignment, with sequence similarity indicated by the shading behind the individual amino acids. Below the aligned sequences, residues lining the −1 sub-site are indicated with green triangles, the catalytic nucleophile and acid/base are indicated by magenta stars and tryptophan side chains narrowing the active site structure in *Bo*GH3B are shown with orange circles.

While GH3B proved readily amenably to crystallization, unfortunately, despite intense efforts, a similarly crystallizable form of GH3A could not be produced. The structure of GH3B was determined to 2.3 Å resolution (electronic supplementary material, table S4) by molecular replacement using the coordinates of barley β-glucosidase (PDB ID: 1ex1, see [52]) as the search model. *Bo*GH3B comprises a three-domain architecture, consisting of an N-terminal (TIM) barrel-like domain (residues 26 to 419), a central α/β sandwich domain (residues 420 to 660) and a fibronectin type-III (FN-III)-like domain at the C-terminus (residues 661–782) (figure 5*a*). Structural comparisons using PDBeFold [56] revealed close structural matches to several other GH3 members, the closest match being to a single protomer of a novel homodimeric GH3 identified in a metagenomic analysis of unnamed soil bacteria (PDBs: 3u48 and 3u4a), with RMSDs of 1.22 and 1.21 Å over 742 and 739 residues, respectively. The dimeric organization of this novel enzyme appears potentially important for function, with a large, flexible loop reaching over from one protomer to contact the substrate and fully assemble the active site of the neighbouring molecule. There is no suggestion of such a dimerization occurring for GH3B, which also shows close matches to more typical monomeric family members including the family 3 β-glucosidases from *Thermatoga neapolitana* (PDBs: 2x42 and 2x41 with RMSDs of 1.49 Å and 1.50 Å, respectively, both over 715 residues) [68] and *Hypocrea jecorina/Trichoderma reesei* (PDBs: 4i8d and 3zyz with RMSDs of 1.42 and 1.50 Å over 711 and 713 residues, respectively) [69]. All of these structures share the same three-domain architecture as GH3B, though maximum identity is no more than 36% at the primary sequence level.

*Bo*GH3B was found to co-purify with glucose in its active site (figure 5*b*). This could readily be modelled with a ^4^C_1_ chair conformation, highlighting the position of the −1 sub-site. As is typical for hydrolytic GH3 members, the active site is formed largely by residues from the core TIM barrel, with additional interactions further contributed by loops from the α/β sandwich domain (figure 5*b*). GH3 members are well-known to employ the classical Koshland double-displacement, configuration-retaining mechanism [70]. Within the GH3B active site, putative catalytic nucleophile (Asp314) and acid/base (Glu534) residues can be observed in close proximity to the glucose moiety, poised for nucleophilic attack. Together with residues forming the −1 sub-site, these interactions appear well conserved, and are maintained in several other GH3–glucose complexes [52,68,69]. Away from the −1 sub-site, the exterior surface structure of the GH3B active pocket deviates from the most closely related homologues, presenting as a more closed structure (figure 5*c*) similar to that seen in the distantly related barley β-glucosidase [52]. The barley enzyme shows quite narrow specificity for β-1,3- and β-1,4-linked glucans, while closer overall structural matches to *Bo*GH3B, including the *T. neapolitana* and *H. jecorina* enzymes described above, show much broader activities against β-1,2-, β-1,3-, β-1,4- and β-1,6-linked disaccharides [68,69]. Such promiscuous catalytic functionality has been suggested to result from the more open active site architecture maintained by this group, allowing diverse linkages and longer substrates to be accommodated (figure 5*d*) [68]. GH3B has significant activity for glucose-only oligosaccharides but displays far weaker activity on xyloglucan-derived oligos, which retain their xylose side chains [32]. Similar to barley β-glucosidase, such observations might suggest that the narrowing of the active site cleft could be responsible for the high specificity of *Bo*GH3B towards β-1,4-linked glucans.

Analysis of residues forming the GH3B +1 sub-site reveals more discernable differences between the two paralogous GH3 members in the *Bo*XyGUL. Sequence analysis suggests poor conservation of two aromatic residues, Trp315 and Trp458 (BoGH43B numbering), which through π-stacking interactions appear to form the narrow GH3B +1 sub-site. Although the equivalent to Trp315 is maintained in GH3A (Trp274), an equivalent to Trp458 appears absent. We hypothesize therefore that GH3A may present a more open active site architecture, leading to a similar rationale in the presence of two GH3 genes to that described above for the *Bo*XyGUL GH43 paralogues. The closed active site pocket in GH3B appears to result in higher affinity interactions with longer ‘cello-oligosaccharides’, suggesting that, as for the two *Bo*GH43 members, subtle differences in the active site architecture might confer adaptations to specific substrates. Again, such a proposal would thus provide a reasonable molecular basis for the maintenance of two highly similar genes in the same operon.

## 4. Conclusion

The absence, within the human genome, of genes encoding enzymes able to metabolize a significant proportion of the complex polysaccharides present in our own diet has thrown into sharp relief the importance of our internal microbial ecosystems [6,71]. The capacity of the gut microbiota to utilize these large, intractable molecules dictates both the composition and correct functioning of this large non-somatic dietary organ, and as such has a direct and crucial impact upon the health of the human host [72]. Recent systems biology approaches have highlighted the many niche roles played by diverse bacteria within the human microbiota [36–39]. While genomics and metagenomics initiatives continue apace, generating increasing amounts of sequence data, further approaches linking sequence data to biological function are essential to understanding the adaptations of individual species that allows them to fulfil their symbiotic role within the human digestive system. Xyloglucan degradation is a niche occupied primarily by the Bacteroidetes, and we have previously highlighted the importance of the specific XyGUL encoded by *B. ovatus* to allow this bacterium to compete for nutrients [32]. Central to this analysis was the tertiary structural characterization of the vanguard endo-xyloglucanase, *Bo*GH5, that catalyses the first backbone hydrolysis step required for xyloglucan polysaccharide metabolism. Recently, we have revealed the key role that two cell-surface glycan-binding proteins (SGBPs) encoded by the XyGUL play in XyG utilization through combined genetic, biophysical and crystallographic analyses [33].

Here, we have significantly extended our knowledge of the structural biology of the XyGUL through crystallography of several exo-glycosidases encoded by the *Bo*XyGUL. This analysis provided insight into the structural features within these enzymes that allow them to interact with and degrade their xyloglucan oligosaccharide substrates. Furthermore, our analysis highlights differences in the structures of two GH43 proteins, which display similar biochemical properties but are maintained within the operon nonetheless. Such observations suggest that these paralogues may play subtly different roles during the degradation of xyloglucans from different sources, or may function most optimally at different stages in the catabolism of XyGOs, for example before or after hydrolysis of certain side-chain moieties. While we were unable to determine a structure for *Bo*GH3A, our structural and sequence analysis of *Bo*GH3B has also allowed us to highlight further potential differences between these two enzymes encoded by the operon. Together with existing biochemical data, our analyses of the three-dimensional structures, and various enzyme-inhibitor complexes, of *Bo*GH31, *Bo*GH43A, *Bo*GH43B and *Bo*GH3B provide molecular-level insight into the stepwise breakdown of xyloglucan by the *Bo*XyGUL. Characterization of key adaptions within these enzymes provides a firm rationale for alternate specificities for XyGOs that may also allow for more efficient degradation of xyloglucan from different sources within the gut.

## Supplementary Material

Supplementary Information

## Data accessibility

All structures and accompanying structure factors have been deposited with the Protein Data Bank (PDB) with accession codes 5JOU, 5JOV, 5JOW, 5JOX, 5JOY, 5JOZ and 5JP0. Individual ITC thermograms and NMR spectra can be found in the electronic supplementary material.
