# Supplementary material for: Structural dissection of a complex Bacteroides ovatus gene locus conferring xyloglucan metabolism in the human gut
Source: Open Biol. 2016 Jul 27;6(7):160142. doi: 10.1098/rsob.160142 (PMC4967831; doi:10.1098/rsob.160142)
Supplement: Supplementary Information [file rsob160142supp1.docx]

# Supporting Information for:

# Structural Dissection of the *Bacteroides ovatus* xyloglucan PUL

**Glyn R Hemsworth^1¶^, Andrew J Thompson^1¶^, Judith Stepper^1^, Łukasz F Sobala^1^, Travis Coyle^2^, Johan Larsbrink^3,4^, Oliver Spadiut^3,5^, Ethan Goddard-Borger^6^, Keith A Stubbs^2^, Harry Brumer^3,4,^*, Gideon J Davies^1,^***

*^1^ Department of Chemistry, York Structural Biology Laboratory, The University of York, Heslington York YO10 5DD U.K.*

*^2^ School of Chemistry and Biochemistry, The University of Western Australia, Crawley, WA 6009, Australia*

*^3^ Division of Glycoscience, School of Biotechnology, Royal Institute of Technology (KTH), AlbaNova University Centre, 106 91 Stockholm, Sweden*

*^4^ Michael Smith Laboratories and Department of Chemistry, University of British Columbia, 2185 East Mall, Vancouver, BC, V6T 1Z4, Canada*

*^5^ Wallenberg Wood Science Center, Royal Institute of Technology (KTH), Teknikringen 56-58, 100 44 Stockholm, Sweden*

*^6^ The Walter and Eliza Hall Institute of Medical Research, 1G Royal Parade, Parkville VIC 3052, Australia*

* to whom correspondence may be addressed

¶ These authors contributed equally to this work.

**Table of Contents.**

Figure S1. Stereo views of (A) the *Bo*GH3B and (B) the *Tn*GH3 active site pocket openings 3

Figure S2. Structure based sequence alignment of *Bo*GH3B with other GH3 family members in the pdb 4-5

Table S1. *Bo*GH31 Data collection and refinement statistics 6

Table S2. *Bo*GH43A Data collection and refinement statistics 7

Table S3. *Bo*GH43B Data collection and refinement statistics 8

Table S4. *Bo*GH3B Data collection and refinement statistics 9

NMR Spectra from Synthesis of Arabinofuranosidase inhibitors 10-14

ITC Data for AraDNJ binding to BoGH43A 15

ITC Data for AraDNJ binding to BoGH43A 16


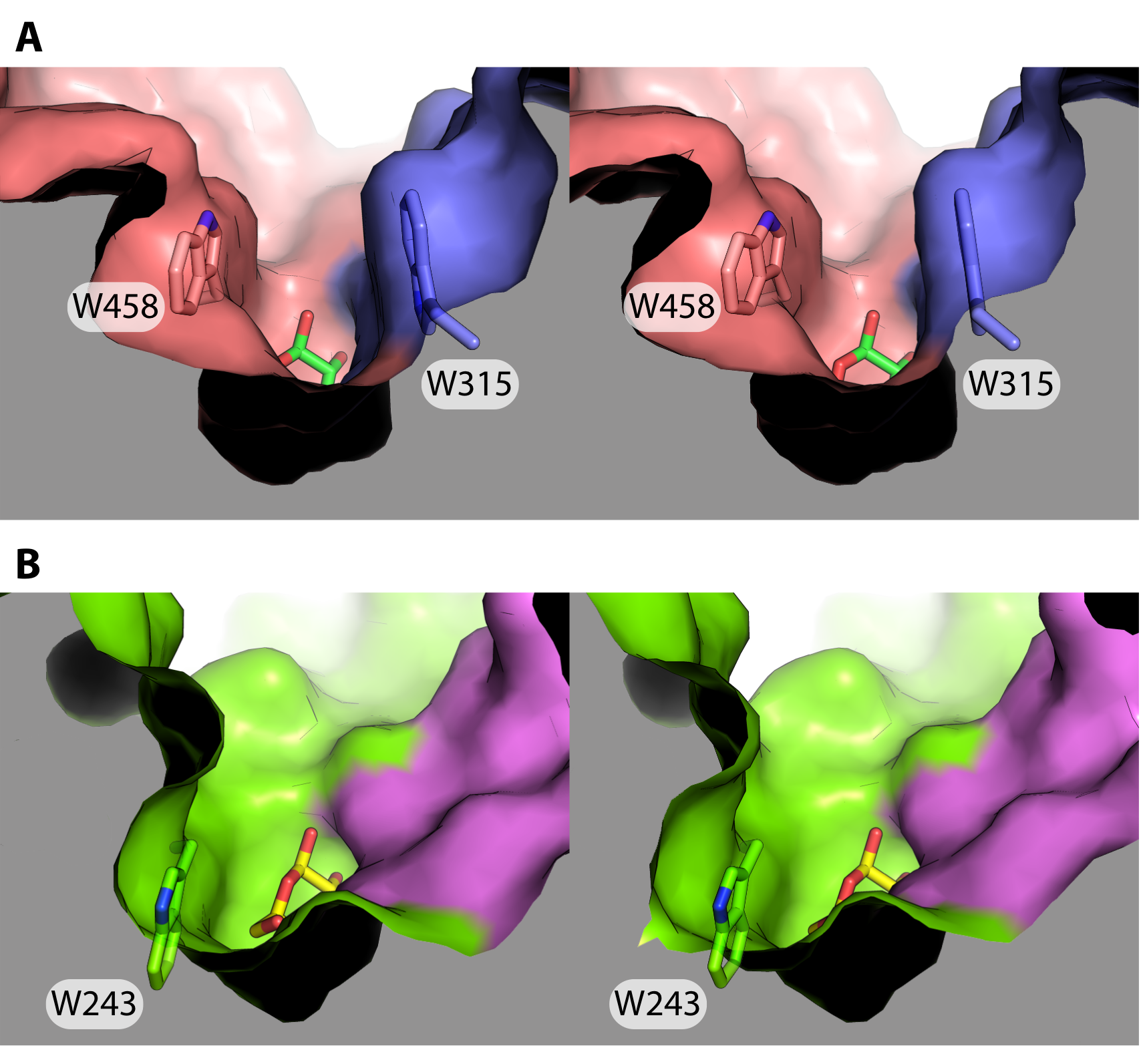


**Figure S1. Stereo views of (A) the *Bo*GH3B and (B) the *Tn*GH3 active site pocket openings.** W458 and W315 can be seen to form a more closed active site structure in *Bo*GH43B in comparison to that seen in *Tn*GH3.


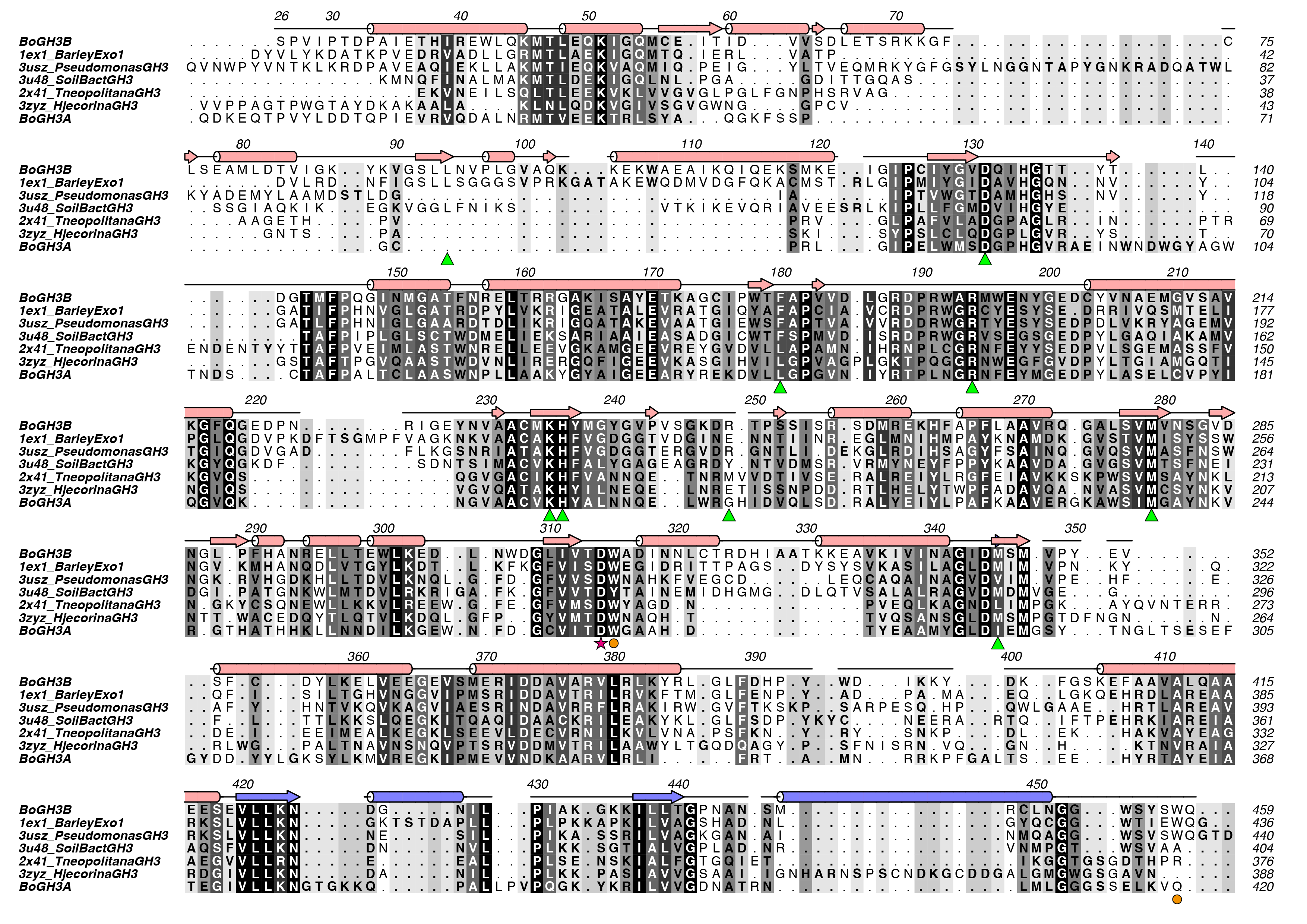

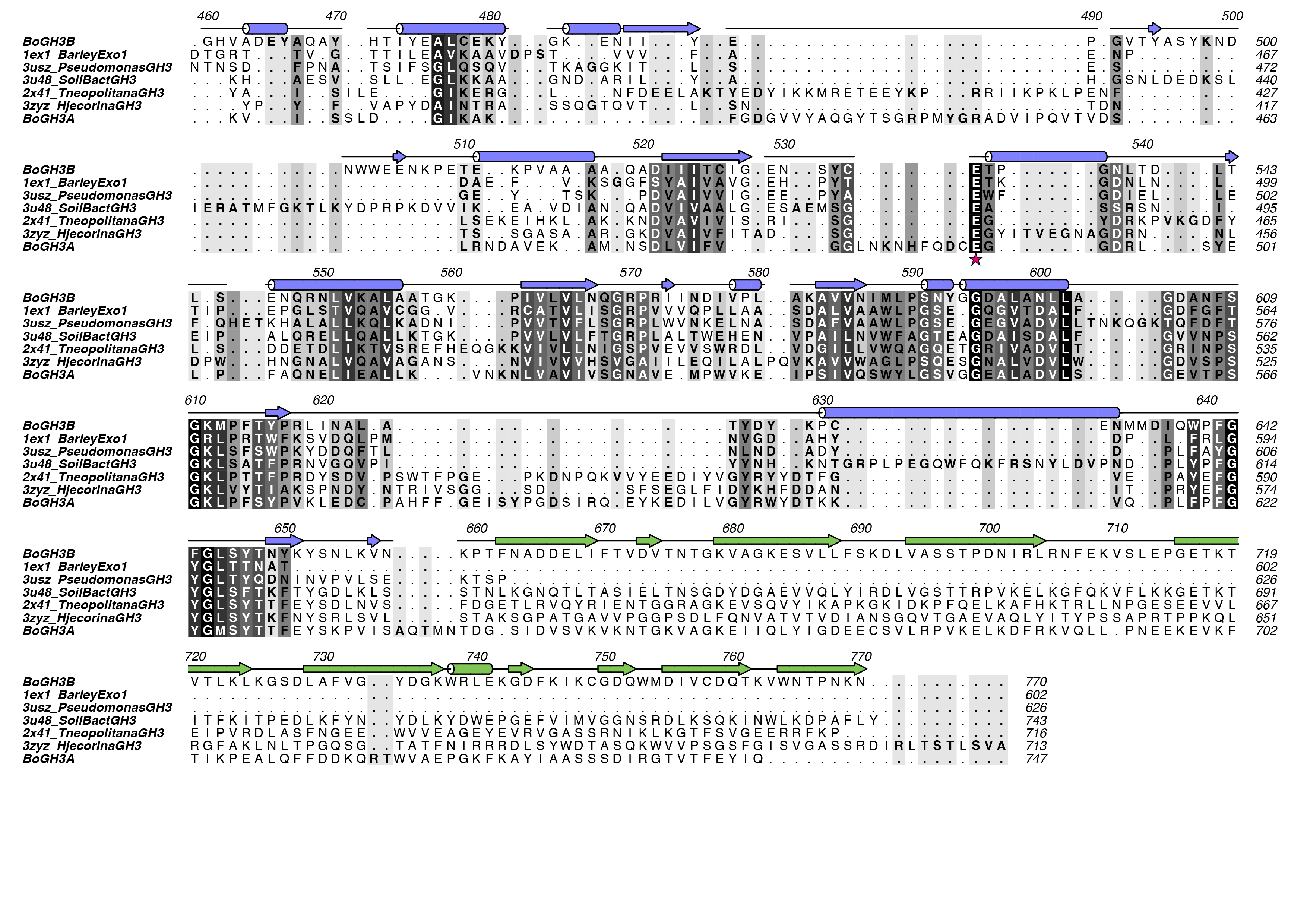


**Figure S2. Structure based sequence alignment of *Bo*GH3B with other GH3 family members in the pdb.** The secondary structure elements and residue numbers from BoGH3B are indicated along the top of the alignment, with sequence similarity indicated by the shading behind the individual amino acids. Below the aligned sequences, residues lining the -1 sub-site are indicated with green triangles, the catalytic nucleophile and acid/base are indicated by magenta stars and tryptophan side chains narrowing the active site structure in BoGH3B are shown with orange circles.

Table S1. *Bo*GH31 Data collection and refinement statistics

|  | Apo | 5F-IdoF |
| --- | --- | --- |
| **Data collection** |  |  |
| Space group | *P*2_1_2_1_2_1_ | *P*2_1_2_1_2_1_ |
| Cell dimensions |  |  |
| *a*, *b*, *c* (Å) | 76.9, 109.0, 145.1 | 76.9, 108.4, 144.9 |
| α, β, γ (°) | 90.0, 90.0, 90.0 | 90.0, 90.0, 90.0 |
| Resolution (Å) | 48.35 - 1.50 (1.53 - 1.50)* | 48.28 - 1.50 (1.52 - 1.50) |
| *R*_merge_ | 0.124 (1.09) | 0.089 (0.91) |
| *R_pim_* | 0.07 (0.85) | 0.05 (0.62) |
| *CC(1/2)* | 0.997 (0.37) | 0.999 (0.60) |
| *I* / σ*I* | 12.4 (1.0) | 12.2 (1.6) |
| Completeness (%) | 96.7 (74.5) | 99.7 (94.0) |
| Redundancy | 7.5 (4.2) | 7.8 (5.4) |
|  |  |  |
| **Refinement** |  |  |
| Resolution (Å) | 1.50 | 1.50 |
| No. reflections | 187821 | 193532 |
| *R*_work_ / *R*_free_ | 0.16/0.18 | 0.11/0.15 |
| *B*-factors (Å^2^) |  |  |
| Protein | 13.1 | 18.5 |
| Ligand | n/a | 13.6 |
| Ion/solvent | 23.4 | 36.4 |
| Water | 32.6 | 39.2 |
| R.m.s. deviations |  |  |
| Bond lengths (Å) | 0.010 | 0.010 |
| Bond angles (°) | 1.41 | 1.37 |
| PDB ID | 5JOU | 5JOV |

*Values in parentheses are for highest-resolution shell.

Table S2. *Bo*GH43A Data collection and refinement statistics

|  | Apo | Ara-DNJ | Ara-LOG |
| --- | --- | --- | --- |
| **Data collection** |  |  |  |
| Space group | *P*2_1_22_1_ | *P*2_1_22_1_ | *P*2_1_22_1_ |
| Cell dimensions |  |  |  |
| *a*, *b*, *c* (Å) | 75.4, 91.2, 156.9 | 75.8, 91.6, 154.4 | 75.6, 91.5, 154.2 |
| α, β, γ (°) | 90.0, 90.0, 90.0 | 90.0, 90.0, 90.0 | 90.0, 90.0, 90.0 |
| Resolution (Å) | 46.69 - 1.60 (1.63 - 1.60)* | 46.58 - 1.80 (1.83 - 1.80) | 46.49 - 1.90 (1.93 - 1.90) |
| *R*_merge_ | 0.092 (1.05) | 0.128 (1.14) | 0.17 (1.44) |
| *R_pim_* | 0.050 (0.59) | 0.073 (0.68) | 0.097 (0.85) |
| *CC(1/2)* | 0.999 (0.68) | 0.994 (0.64) | 0.994 (0.43) |
| *I* / σ*I* | 14.5 (1.9) | 8.5 (1.2) | 8.6 (1.6) |
| Completeness (%) | 100.0 (100.0) | 99.9 (100.0) | 99.8 (99.6) |
| Redundancy | 8.1 (8.0) | 7.5 (7.2) | 7.9 (7.7) |
|  |  |  |  |
| **Refinement** |  |  |  |
| Resolution (Å) | 1.60 | 1.80 | 1.90 |
| No. reflections | 142867 | 100168 | 84685 |
| *R*_work_ / *R*_free_ | 0.13/0.17 | 0.18/0.22 | 0.18/0.22 |
| *B*-factors (Å^2^) |  |  |  |
| Protein | 18.5 | 26.7 | 23.9 |
| Ligand | n/a | 23.9 | 27.7 |
| Ion/Solvent | 32.5 | n/a | 43.6 |
| Water | 35.2 | 36.3 | 33.3 |
| R.m.s. deviations |  |  |  |
| Bond lengths (Å) | 0.009 | 0.009 | 0.009 |
| Bond angles (°) | 1.37 | 1.45 | 1.38 |
| PDB ID | 5JOW | 5JOX | 5JOY |

*Values in parentheses are for highest-resolution shell.

Table S3. *Bo*GH43B Data collection and refinement statistics

|  | BoGH43B |
| --- | --- |
| **Data collection** |  |
| Space group | C2 |
| Cell dimensions |  |
| *a*, *b*, *c* (Å) | 91.5, 71.4, 160.3 |
| α, β, γ (°) | 90.0, 94.8, 90.0 |
| Resolution (Å) | 52.19 - 2.28 (2.34 - 2.28) * |
| *R*_merge_ | 0.092 (0.624) |
| *R_pim_* | 0.079 (0.529) |
| *CC(1/2)* | 0.996 (0.735) |
| *I* / σ*I* | 11.1 (2.3) |
| Completeness (%) | 99.6 (99.5) |
| Redundancy | 4.1 (4.2) |
|  |  |
| **Refinement** |  |
| Resolution (Å) | 52.19 - 2.28 |
| No. reflections | 44617 |
| *R*_work_ / *R*_free_ | 0.192/0.250 |
| *B*-factors (Å^2^) |  |
| Protein | 35.6 |
| Ligand/ion | 33.3 |
| Water | 30.5 |
| R.m.s. deviations |  |
| Bond lengths (Å) | 0.008 |
| Bond angles (°) | 1.32 |
| PDB ID | 5JOZ |

*Values in parentheses are for highest-resolution shell.

Table S4. *Bo*GH3B Data collection and refinement statistics

|  | GH3B |
| --- | --- |
| **Data collection** |  |
| Space group | P2_1_2_1_2_1_ |
| Cell dimensions |  |
| *a*, *b*, *c* (Å) | 97.2, 104.1, 160.4 |
| α, β, γ (°) | 90.0, 90.0, 90.0 |
| Resolution (Å) | 47.57 - 2.30 (2.35 - 2.30) * |
| *R*_merge_ | 0.151 (0.923) |
| *R_pim_* | 0.083 (0.496) |
| *CC(1/2)* | 0.995 (0.777) |
| *I* / σ*I* | 11.4 (2.1) |
| Completeness (%) | 99.9 (100.0) |
| Redundancy | 8.1 (8.5) |
|  |  |
| **Refinement** |  |
| Resolution (Å) | 87.31 - 2.30 |
| No. reflections | 69286 |
| *R*_work_ / *R*_free_ | 0.177/0.211 |
| *B*-factors (Å^2^) |  |
| Protein | 35.8 |
| Ligand/ion | 31.9/26.4 |
| Water | 28.4 |
| R.m.s. deviations |  |
| Bond lengths (Å) | 0.010 |
| Bond angles (°) | 1.41 |
| PDB ID | 5JP0 |

*Values in parentheses are for highest-resolution shell.

NMR Spectra from Synthesis of Arabinofuranosidase inhibitors

^1^H of (*Z*)-2,3,5-Tri-*O*-acetyl-L-arabinonhydroximo-1,4-lactone **3**


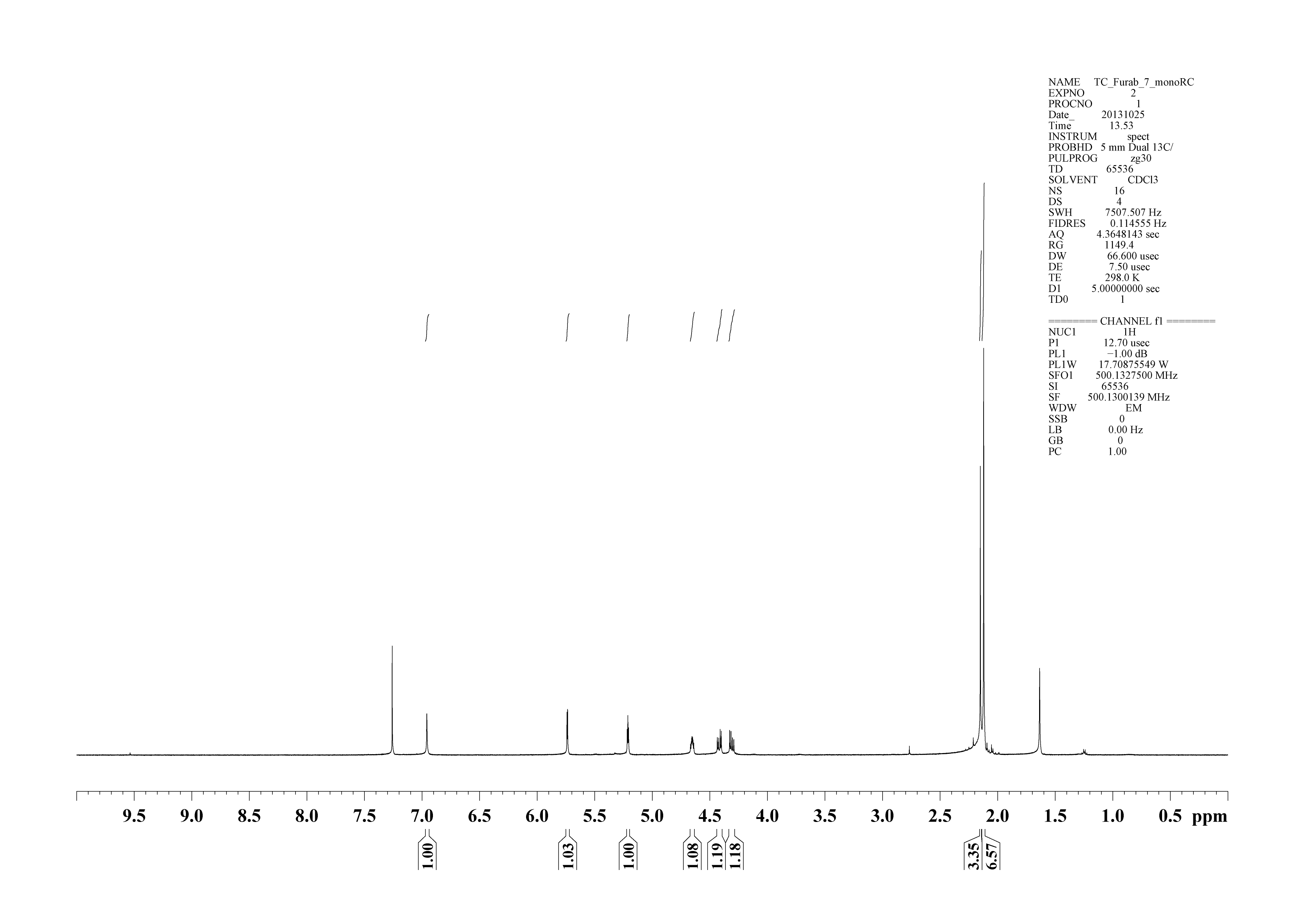


^13^C of (*Z*)-2,3,5-Tri-*O*-acetyl-L-arabinonhydroximo-1,4-lactone **3**


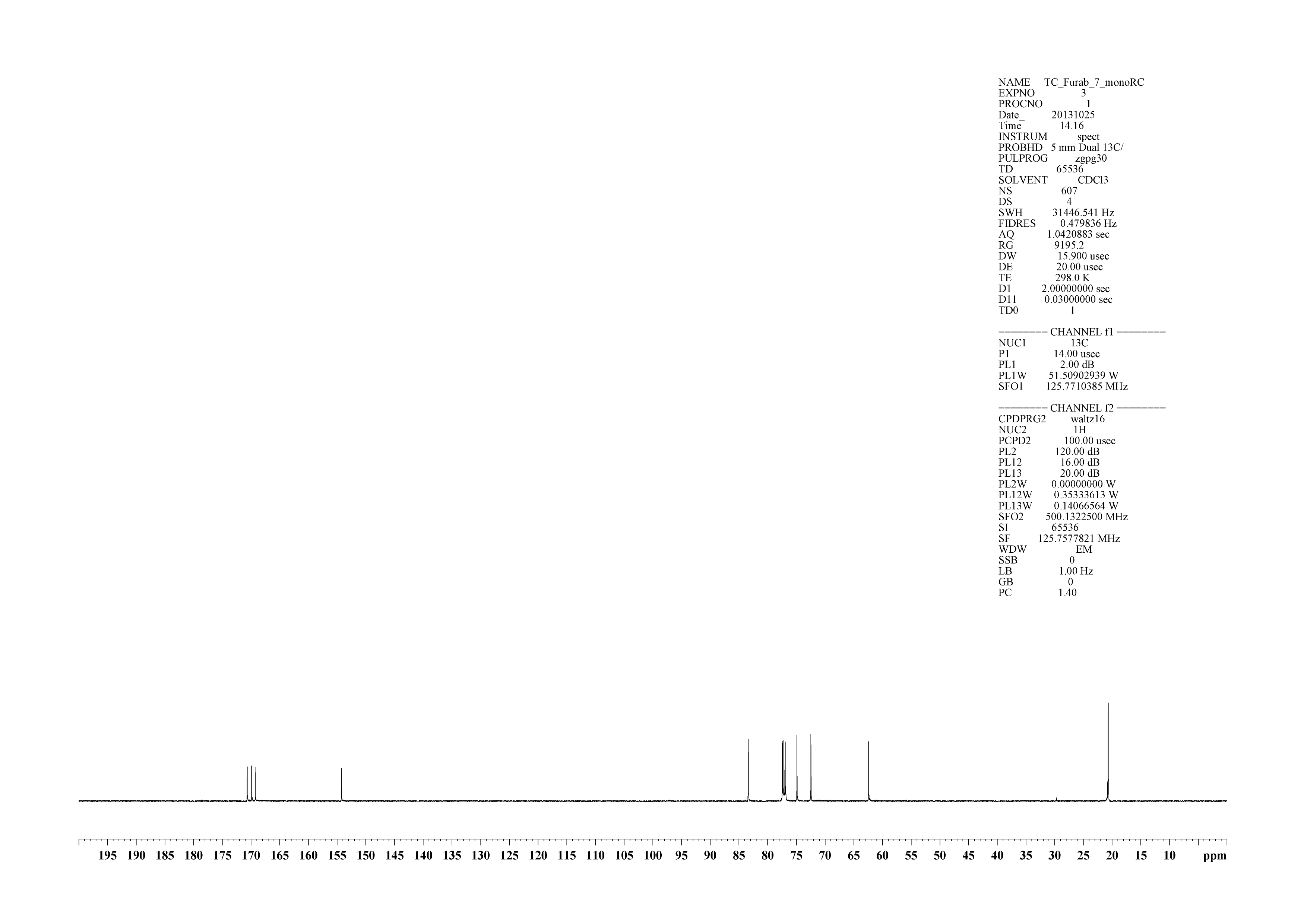


^1^H of (*Z*)-L-arabinonhydroximo-1,4-lactone (AraLOG)


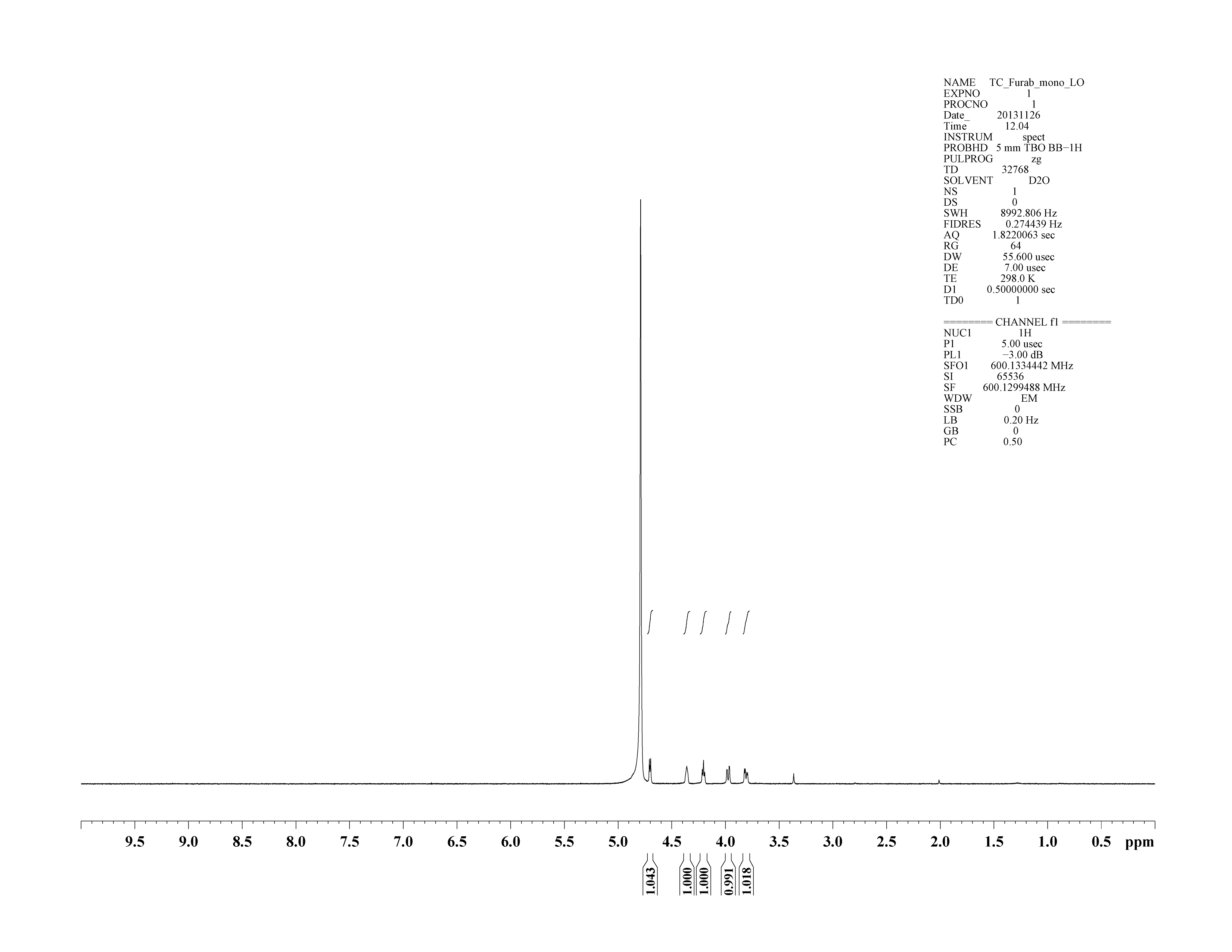


^13^C of (*Z*)-L-arabinonhydroximo-1,4-lactone (AraLOG)


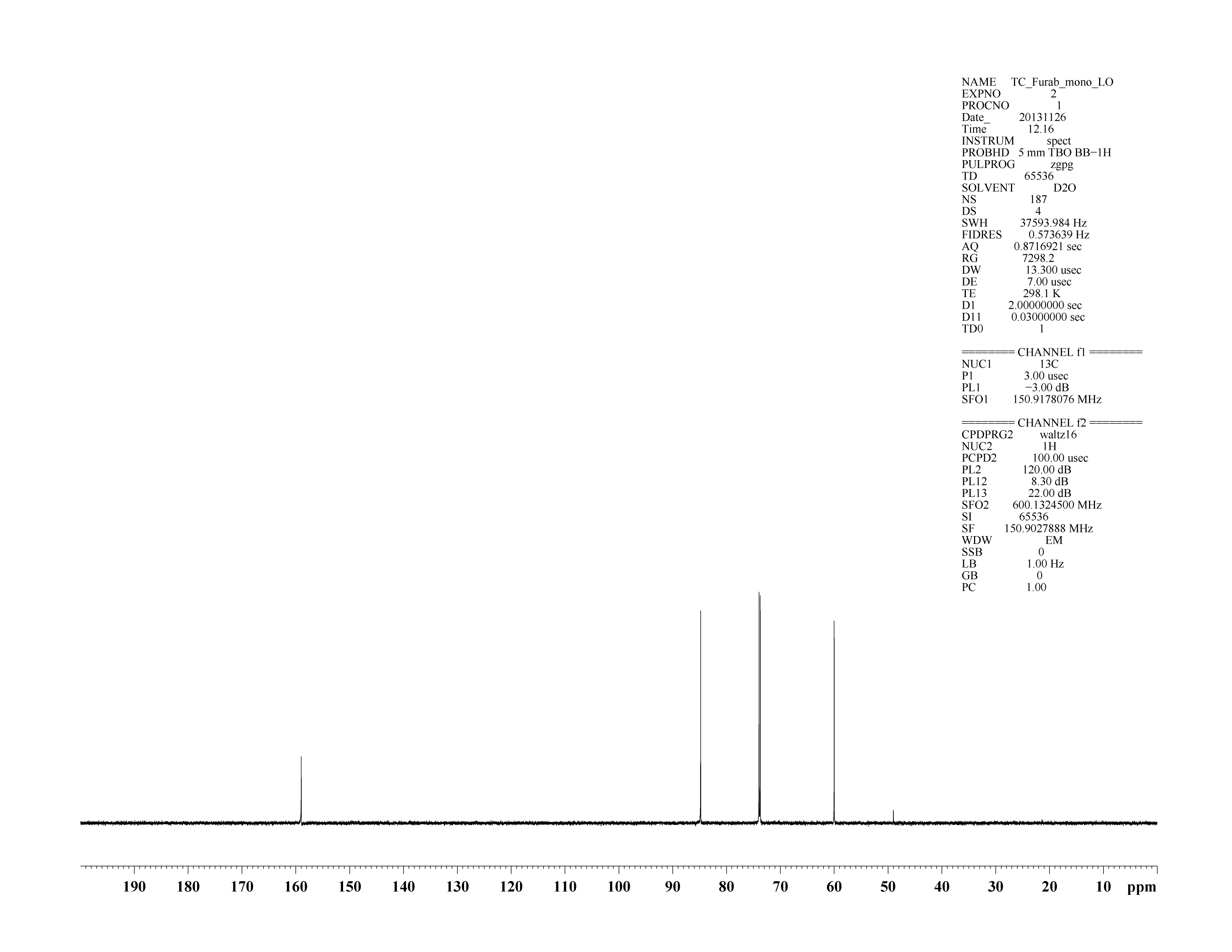


^1^H of (*Z*)-*O*-(2,3,5-Tri-*O*-acetyl-L-arabinosylidene)amino N-phenylcarbamate **4**


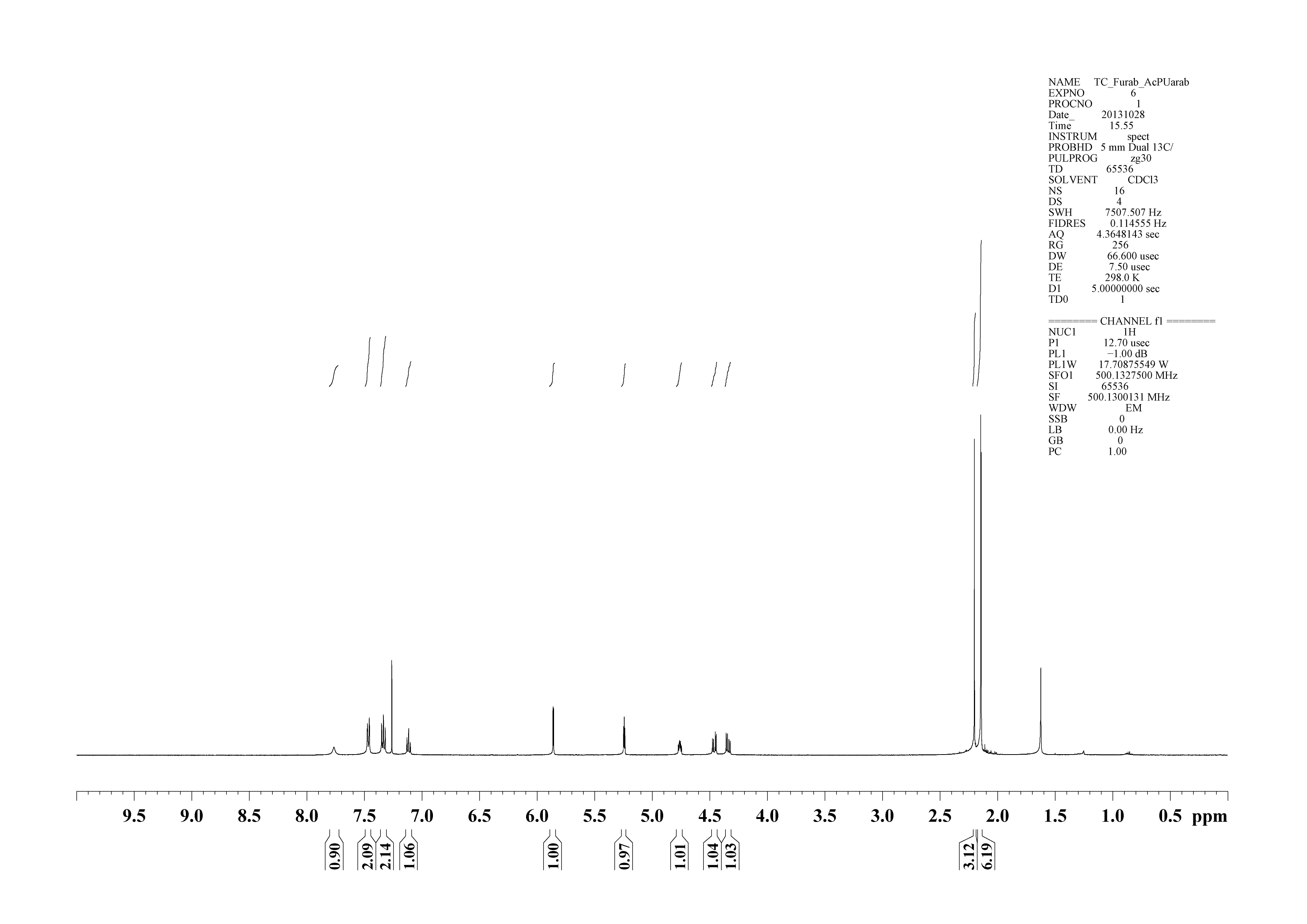


^13^C of (*Z*)-*O*-(2,3,5-Tri-*O*-acetyl-L-arabinosylidene)amino N-phenylcarbamate **4**


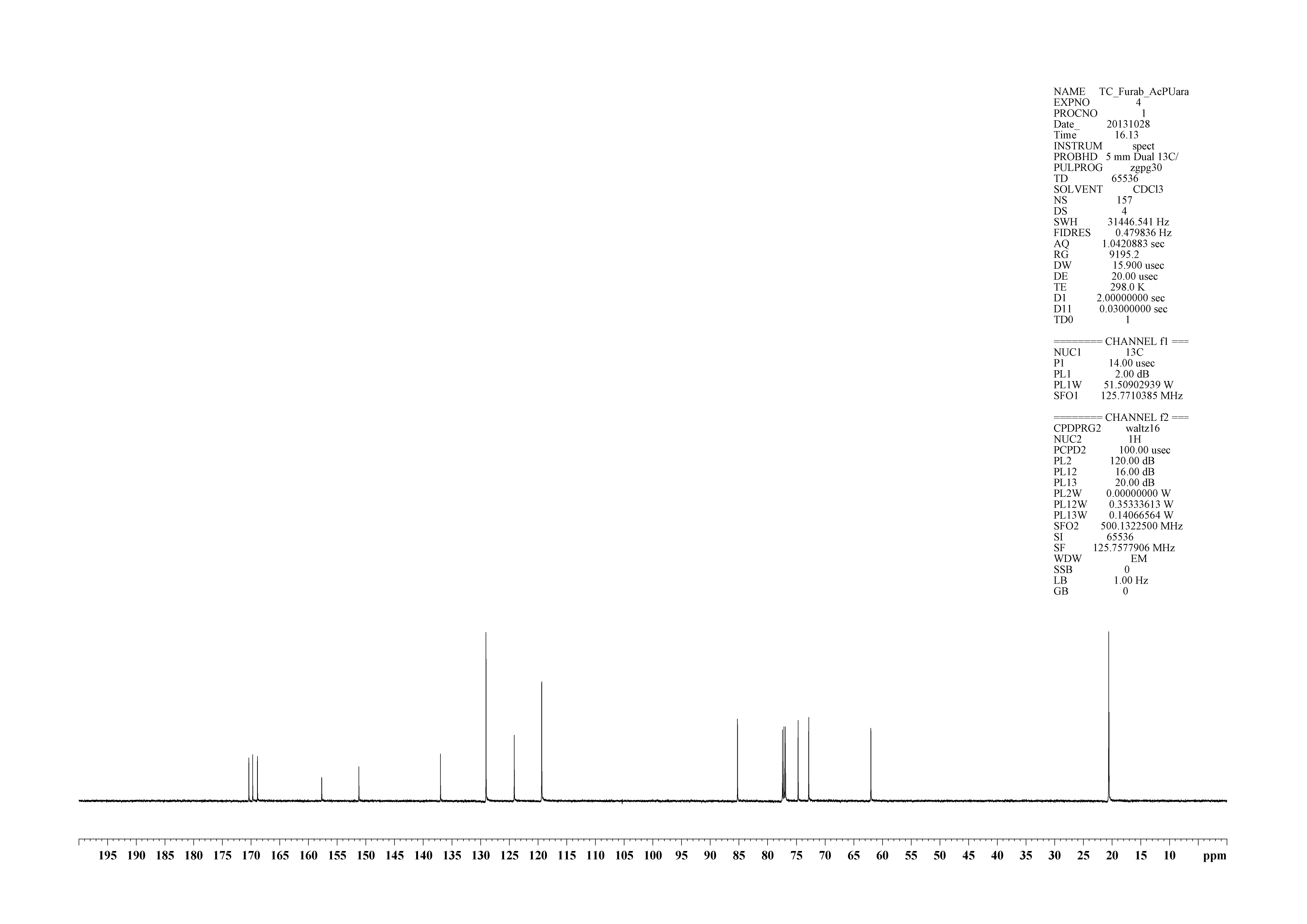


^1^H of (*Z*)-*O*-(L-arabinosylidene)amino N-phenylcarbamate (AraPUG)


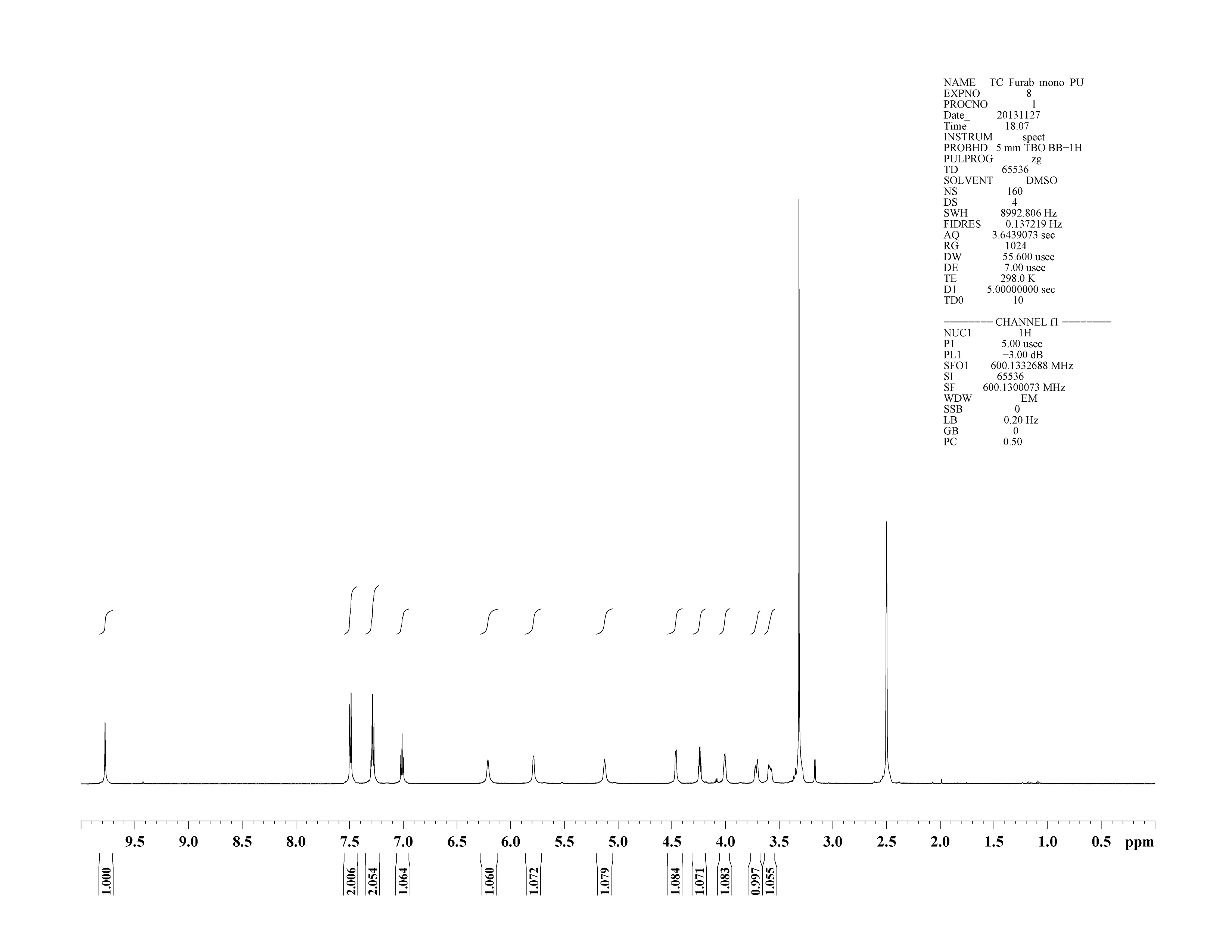


^13^C of (*Z*)-*O*-(L-arabinosylidene)amino N-phenylcarbamate (AraPUG)


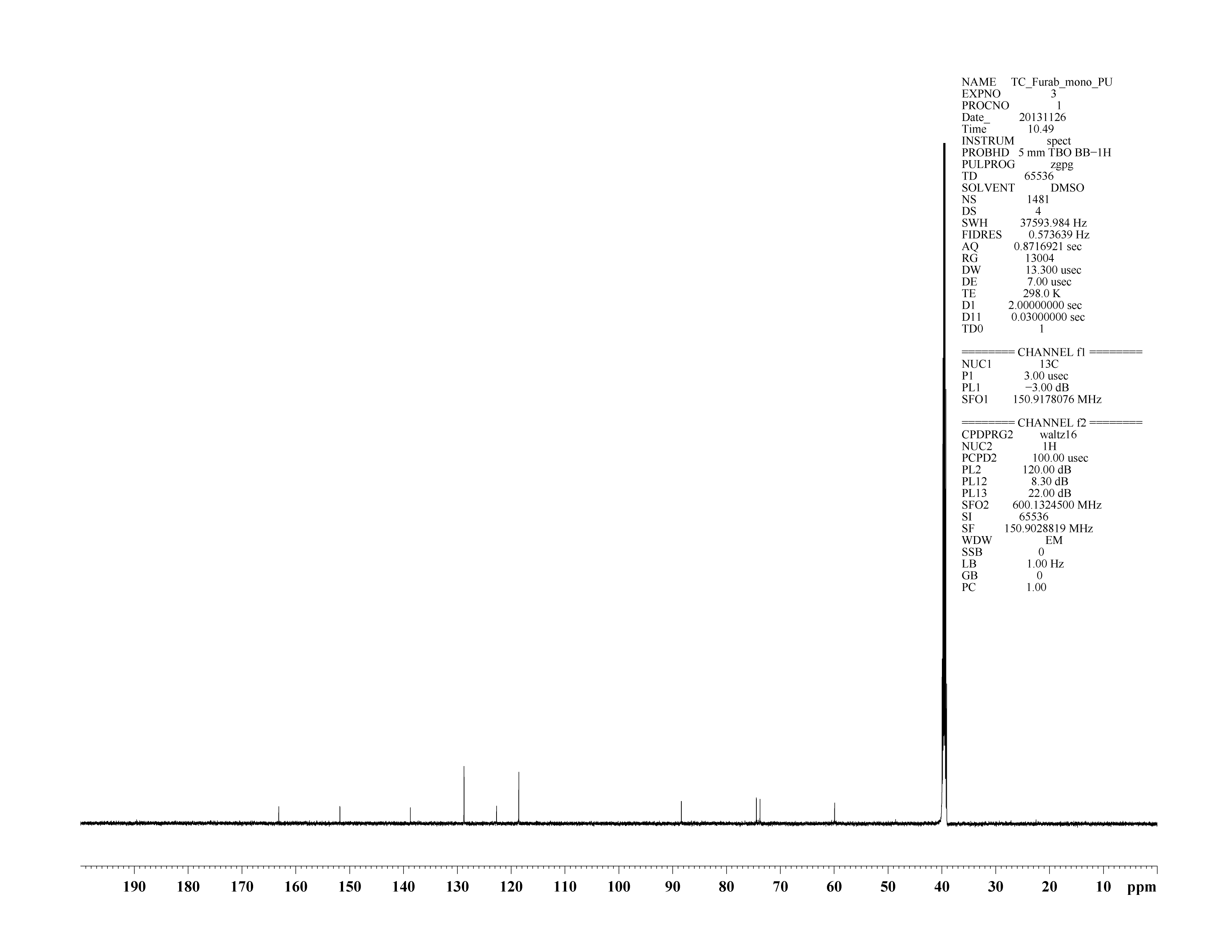


ITC Data for Binding of AraDNJ to BoGH43A.

(**A-C**) ITC thermograms showing AraDNJ binding to BoGH43A in triplicate. (**D**) Results of fitting a single site binding model to each ITC run using Origin 7.

ITC Data for Binding of AraDNJ to BoGH43B.

(**A-C**) ITC thermograms showing AraDNJ binding to BoGH43B in triplicate. (**D**) Results of fitting a single site binding model to each ITC run using Origin 7.
